# Atomic Dispersed Hetero-Pairs for Enhanced Electrocatalytic CO_2_ Reduction

**DOI:** 10.1007/s40820-023-01214-2

**Published:** 2023-11-06

**Authors:** Zhaoyong Jin, Meiqi Yang, Yilong Dong, Xingcheng Ma, Ying Wang, Jiandong Wu, Jinchang Fan, Dewen Wang, Rongshen Xi, Xiao Zhao, Tianyi Xu, Jingxiang Zhao, Lei Zhang, David J. Singh, Weitao Zheng, Xiaoqiang Cui

**Affiliations:** 1 State Key Laboratory of Automotive Simulation and Control, School of Materials Science and Engineering, Key Laboratory of Automobile Materials of MOE, Electron Microscopy Center, Jilin University, Changchun, 130012 People’s Republic of China; 2 Key Laboratory of Photonic and Electronic Bandgap Materials, Ministry of Education, and College of Chemistry and Chemical Engineering, Harbin Normal University, Harbin, 150025 People’s Republic of China; 3 College of Chemistry, Jilin University, Changchun, 130012 People’s Republic of China; 4 Department of Physics and Astronomy and Department of Chemistry, University of Missouri, Columbia, MO 65211 USA

**Keywords:** CO_2_ reduction reaction, Atomic dispersed catalyst, Hetero-diatomic pair, Ad-desorption energy, Linear scaling relation

## Abstract

Electrochemical carbon dioxide reduction reaction (CO_2_RR) involves a variety of intermediates with highly correlated reaction and ad-desorption energies, hindering optimization of the catalytic activity. For example, increasing the binding of the *COOH to the active site will generally increase the *CO desorption energy. Breaking this relationship may be expected to dramatically improve the intrinsic activity of CO_2_RR, but remains an unsolved challenge. Herein, we addressed this conundrum by constructing a unique atomic dispersed hetero-pair consisting of Mo-Fe di-atoms anchored on N-doped carbon carrier. This system shows an unprecedented CO_2_RR intrinsic activity with TOF of 3336 h^−1^, high selectivity toward CO production, Faradaic efficiency of 95.96% at − 0.60 V and excellent stability. Theoretical calculations show that the Mo-Fe diatomic sites increased the *COOH intermediate adsorption energy by bridging adsorption of *COOH intermediates. At the same time, d-d orbital coupling in the Mo-Fe di-atom results in electron delocalization and facilitates desorption of *CO intermediates. Thus, the undesirable correlation between these steps is broken. This work provides a promising approach, specifically the use of di-atoms, for breaking unfavorable relationships based on understanding of the catalytic mechanisms at the atomic scale.

**Supplementary Information:**

The online version contains supplementary material available at 10.1007/s40820-023-01214-2.

## Highlights

- A unique atomic dispersed hetero-pair was successfully synthesized, consisting of Mo-Fe di-atoms anchored on N-doped carbon carrier.
- This strategy breaks the linear scaling relationships of electrocatalytic CO_2_ reduction by simultaneously regulating the *COOH adsorption energy and *CO desorption energy.
- The as-prepared MoFe–N–C exhibits excellent performance for CO_2_RR to CO with a high turnover frequency (TOF) of 3336.21 h^−1^, CO Faradaic efficiency (FE_CO_) of 95.96% at − 0.60 V (versus RHE) and outstanding stability.

**Supplementary Information:**

The online version contains supplementary material available at 10.1007/s40820-023-01214-2.

## Graphical abstract

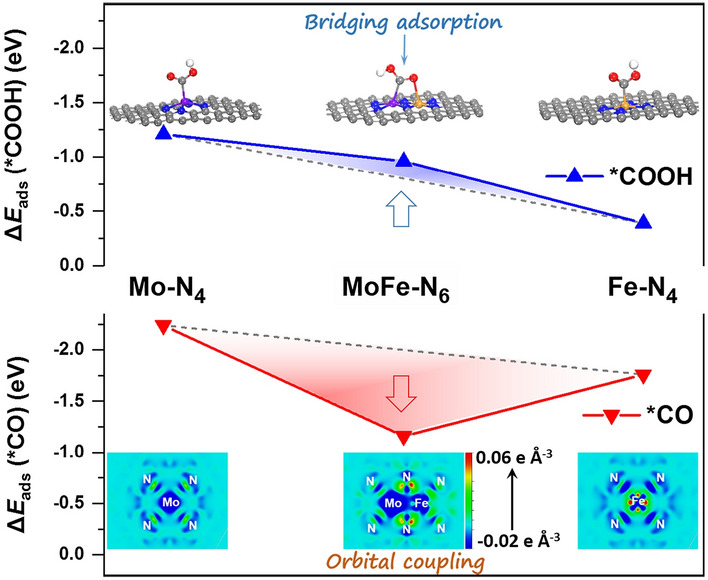

## Introduction

The electrochemical carbon dioxide reduction reaction (CO_2_RR), converting CO_2_ into valuable chemicals, using electrocatalysts and electricity that can be generated from sustainable energy sources provides an effective strategy for achieving carbon neutrality [1–5]. However, this technology is limited by high overpotentials, sluggish electron transfer kinetics and poor selectivity of the catalysts [6–9]. The CO_2_RR process involves a well-established multistep proton–electron transfer mechanism with multiple reaction intermediates, in particular *COOH, *CHO and *CO [10–13]. The catalytic activity is limited by the strong correlations between the adsorption energies of intermediates, referred to as the linear scaling relationship. Importantly, this hinders the design of catalysts due to the competition between optimization of the reaction intermediate formation and that of product desorption [14, 15]. Specifically, for CO_2_ reduction to CO, the active site should bind strongly with the *COOH intermediate for the first electron transfer to CO_2_ molecule [16, 17]. Conversely, the adsorption energy of *CO intermediate should be reduced to facilitate the desorption of final products [18]. However, the linear scaling relationship prevents optimization of both [19]. Several catalyst design tactics for overcoming this have been proposed and tested to overcome this limitation with positive results. These include surface functionalization [20], multi-metallic alloying [21], strain/defect engineering [21, 22], and morphology control [23]. In addition, regulating the concentration of alkali metal cations in the electrolyte has also been explored to break the linear scaling relationship [24, 25]. Nevertheless, breaking the linear scaling relationship is still experimentally and theoretically challenging, with the result that the performance of existing electrocatalysts for this important reaction remains limited.

Recently, atomic dispersed single-atom catalysts (SACs) and diatomic site catalysts (DASCs) have shown promise for electrocatalytic CO_2_RR due to their maximum metal atom utilization, unsaturated coordination environments, tunable electronic states and exposed active sites [26–28]. They are also recognized as ideal model systems for studying the catalytic mechanism at atomic level [29, 30]. However, the linear scaling relationship has also constrained improvement of their performance. For instance, atomic dispersed Ni sites provides superior ability to suppress the HER, but suffers from the large onset potential due to the high *COOH formation energy [31–33]; Fe sites show lower onset potential, but the selectivity for electrocatalytic CO_2_RR is limited due to the inhibited desorption of *CO intermediates [34, 35]. Although the catalytic activities can be enhanced by manipulating the coordination environment and charge distribution of metal sites [36–40], there remains the challenge of breaking the linear scaling relationship [34, 35].

Here, we construct a unique atomic dispersed hetero-pair consisting of Mo-Fe di-atoms anchored on a N-doped carbon carrier (MoFe-N–C). The resulting as-prepared catalyst exhibits excellent performance for CO_2_RR to CO with a high turnover frequency (TOF) of 3336.21 h^−1^, CO Faradaic efficiency (FE_CO_) of 95.96% at − 0.60 V (versus RHE) and outstanding stability. Density functional theory (DFT) calculations indicate that the adsorption energy of the *COOH intermediate is increased due to a unique bridge adsorption configuration. Specifically, the C and O atoms of the *COOH intermediate are adsorbed on the Mo and Fe atoms of the Mo-Fe pair, respectively. This increases the electron transfer to *COOH intermediates. At the same time, the desorption energy of the longitudinally adsorbed *CO intermediates is decreased due to the d–d orbital coupling between the Mo-Fe pair. This reduces the tendency of the pair to bind *CO. In particular, this orbital coupling results in electron delocalization and reduces the charge density of metal sites, thus reducing the adsorption energy of CO intermediates that are adsorbed on Fe atoms. Our strategy simultaneously regulates the *COOH adsorption energy and *CO desorption energy. This finally breaks the linear scaling relationships and significantly enhances the catalytic efficiency. This discovery provides a useful strategy for designing efficient electrochemical CO_2_RR catalysts.

## Experimental Section

### Chemicals and Materials

The Zinc (II) nitrate hexahydrate (Zn(NO_3_)_2_·6H_2_O), potassium bicarbonate (KHCO_3_), potassium hydroxide (KOH), potassium chloride (KCl), sodium hydroxide (NaOH), ethanol (C_2_H_6_O), melamine (C_3_H_6_N_6_) and methanol (CH_3_OH) were all purchased from Sinopharm Chemical Reagent Co., China. Iron phthalocyanine (C_32_H_16_FeN_8_) was obtained from Alfa Aesar, the USA. Bis(acetylacetonato) dioxomolybdenum (VI) (C_10_H_14_MoO_6_), 2-methylimidazole (C_4_H_6_N_2_) and iron (III) acetylacetonate (C_15_H_21_FeO_6_) were obtained from Shanghai Aladdin Biochemical Technology Co., Ltd., China. Nafion-ethanol solution was obtained from Adamas-beta Chemical Co., Switzerland. CO_2_ (99.999%) and Ar (99.999%) were purchased from Xin’guang Gas Co., China. Carbon fiber paper was obtained from Avcarb, the USA. 211 Nafion membrane was purchased from Dupont, the USA. All chemicals were commercial and used without further purification. Ultrapure water (18.2 MΩ cm) was used in all experiments.

### Catalyst Synthesis

#### Synthesis of Mo-ZIF-8 and ZIF-8

The In a typical synthesis, bis(acetylacetonato) dioxomolybdenum (6.5 mg, 0.02 mmol) and Zn(NO_3_)_2_·6H_2_O (4.48 g, 16 mmol) were first dissolved in methanol solution (80 mL) by stirring for 20 min. Then, 2-methylimidazole (5.26 g, 64 mmol) was added into 60 mL methanol solution and stirring for 15 min to ensure complete solvation. Afterward, the mixed solution of bis(acetylacetonato) dioxomolybdenum and Zn(NO_3_)_2_ was slowly added to the 2-methylimidazole solution with stirring. Incubate the mixture at room temperature for 20 h to complete the self-assembly process without stirring. Next, the white powder obtained was centrifuged and washed three times with methanol. Finally, white Mo-ZIF-8 powder was obtained after 5 h vacuum drying. ZIF-8 was prepared by the same method as Mo-ZIF-8 except that bis(acetylacetonato) dioxomolybdenum was not added in the preparation process.

#### ***Synthesis of Mo***_***first***_***–N–C, MoFe–N–C and Mo–N-C***

The powder of Mo-ZIF-8 was transferred into a ceramic boat and placed in a tube furnace. The sample was heated to 900 °C for 3 h with a heating rate of 5 °C min^−1^ under flowing N_2_ gas and naturally cooled to room temperature. Then, the sample was heated to 1000 °C for 1 h with a heating rate of 5 °C min^−1^ to obtain Mo_first_-N–C.

Next, 0.10 g Mo_first_–N–C powder was weighed and added to a beaker containing 30 mL methanol solution and ultrasonic treatment for 30 min to make it uniformly dispersed. Then, a certain concentration of iron acetylacetonate methanol solution. The mixed solution was stirred at room temperature for 20 h so that the iron acetylacetonate was fully absorbed by Mo_first_–N–C. The black powder obtained after centrifugal drying. Finally, the powder was heated to 800 °C with a heating rate of 5 °C min^−1^ and kept 2 h under flowing N_2_ gas and then naturally cooled to room temperature. The obtained black powder was labeled MoFe–N–C. Mo–N–C was prepared with the same method as MoFe–N–C except that iron acetylacetonate was not added in the preparation process.

#### Synthesis of Fe–N-C and N–C

Fe–N-C was prepared by the same method as MoFe-N–C, but ZIF-8 was used in the preparation process instead of Mo-ZIF-8 as the precursor. N–C were prepared with the same method as Fe–N–C except that iron acetylacetonate was not added in the preparation process.

### Electrochemical Measurements

The electrochemical measurements were carried out on a CHI 760e electrochemical workstation in a sealed three-electrode configuration cell using carbon paper coated with catalyst as the working electrode, platinum plate as the counter electrode, and Ag/AgCl (saturated KCl) as the reference electrode in 0.5 M KHCO_3_ aqueous electrolyte. Before preparing the working electrode, all the powdered catalysts were carefully ground with a mortar. All catalysts ink was drop-casted onto a carbon paper (2 cm × 3 cm) electrode and dried under an infrared lamp to obtain a working electrode with a catalyst mass loading of 1 mg cm^−2^. The cathodic and anodic compartments were separated by an anion exchange membrane. Before the measurement, the KHCO_3_ aqueous solution was purged by bubbling CO_2_ for 30 min until saturation. During the electrolysis process, CO_2_ gas is always bubbling with a flow rate of 40 sccm and the electrolyte flowing at a flow rate of 60 sccm. After the electrolysis, the CO_2_ reduction products were sampled and analyzed by GC. All potentials reported are with regard to the reversible hydrogen electrode (RHE), calculated using the following equation: E_RHE_ = E_Ag/AgCl_ + 0.197 V + 0.059 V × pH.

## Results and Discussion

### Structural Characterizations

**Fig. 1:**
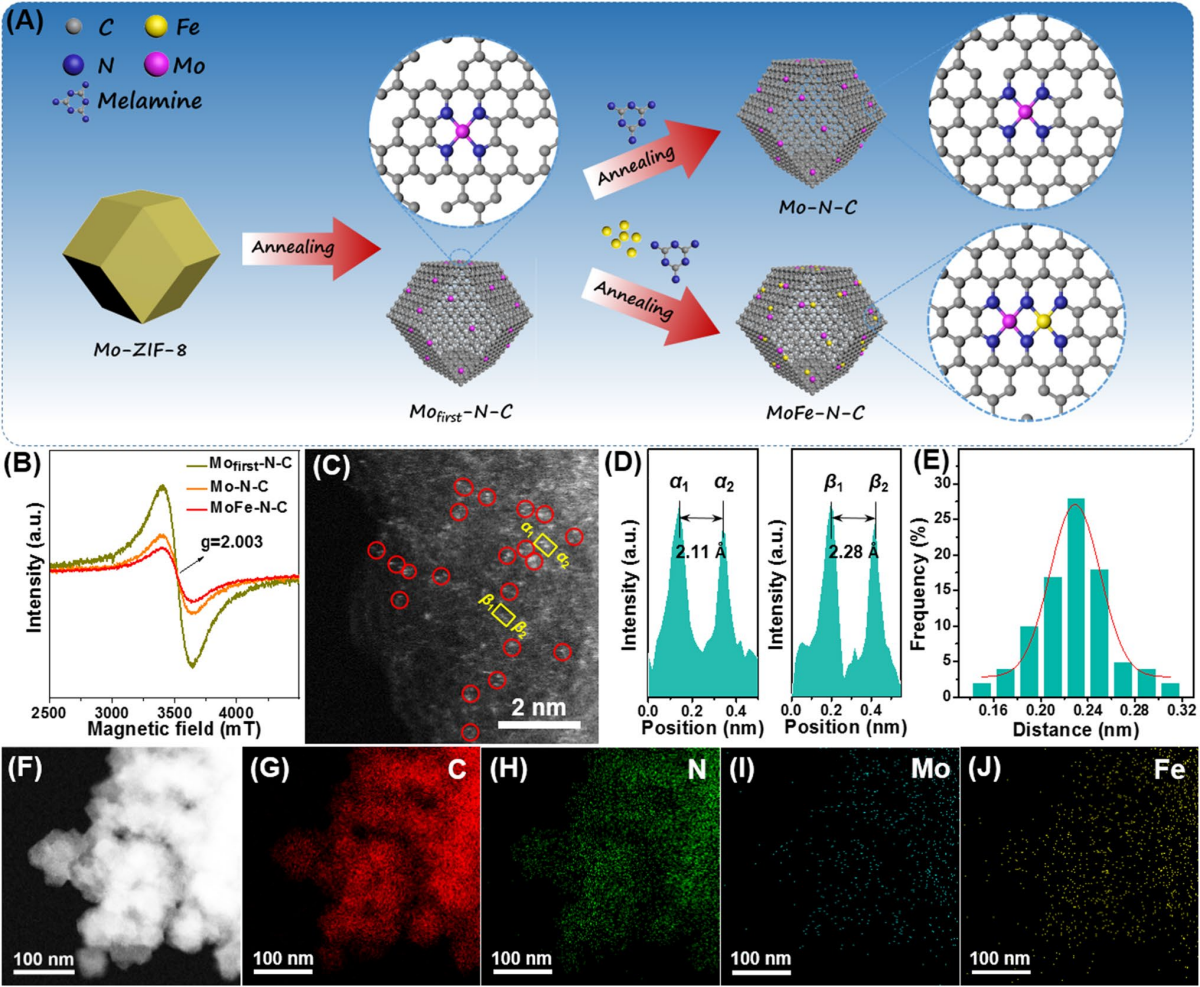
**A** Schematic illustration of the synthesis procedure. **B** EPR spectra of Mo_first_-N–C, Mo–N–C and FeMo–N–C. **C** Aberration-corrected HAADF-STEM image; bimetallic Fe-Mo sites are highlighted by red circles. **D** The intensity profiles obtained from the yellow boxes in (**C**). **E** Statistical Fe-Mo distance in the observed diatomic pairs. **F**–**J** HAADF-STEM image of MoFe–N–C with EDS mappings of individual elements (C, N, Mo and Fe)

The MoFe–N–C catalyst consisting of atomic dispersed Mo-Fe hetero-pairs was synthesized by a modified continuous two-step approach (Fig. 1A) [41]. N–C, Mo–N–C and Fe–N–C catalysts were also prepared by similar methods for comparison (details are in the Supporting Information). The morphology was characterized by scanning electron microscopy (SEM) and transmission electron microscope (TEM). The pristine ZIF-8 and Mo-doped ZIF-8 (Mo-ZIF-8) present a uniform rhombic dodecahedron morphology with diameters of 86.06 ± 6.93 nm and 85.44 ± 6.91 nm, respectively (Fig. S1). The rhombic dodecahedron morphology of Mo_first_–N–C, N–C, Mo–N–C, Fe–N–C and MoFe–N–C is well maintained, but the surface is slightly collapsed and the diameters were reduced, relative to the pristine materials, to 54.07 ± 5.34, 53.08 ± 4.74, 51.85 ± 4.60, 52.31 ± 6.08 and 53.58 ± 5.05 nm, respectively (Figs. S2–S4). No obvious bulk-like metallic phases are observed from HR-TEM for these four amorphous carbon-based catalysts. Brunauer–Emmett–Teller (BET) results show that the catalyst surface areas were relatively reduced after annealing (Fig. S5). Furthermore, the pore size distribution confirms that the microporous structure of N–C, Mo–N–C, Fe–N–C and MoFe–N–C has not been destroyed, which provided a more effective surface area and channels for electrolyte and CO_2_ accessibility. EPR spectroscopy was used to monitor vacancy formation during the preparation as shown in Fig. 1B. The Mo_first_-N–C material presents a Lorentzian line with *g* = 2.003, stemming from the unpaired electrons on the carbon atoms of the aromatic rings [42]. This indicates that a large number of carbon vacancies were formed in Mo_first_-N–C after annealing Mo-ZIF-8 [43, 44]. After further annealing with melamine as N source, the EPR signal of the Mo–N–C decreased significantly. These results show that the annealing process is able to repair the carbon vacancies and improve the carbonization of the carrier and facilitate the electron transfer (Fig. S6). This is further confirmed by Raman spectroscopy (Fig. S7) [41, 45]. Notably, the EPR signal of MoFe–N–C is smaller than that of Mo–N–C, suggesting that the carbon vacancies are further removed by Fe atom anchoring [29].

The structure of the catalysts was investigated by XRD. The characteristic peaks of Mo-ZIF-8 and ZIF-8 are preserved with very little change. This shows that the doping by Mo atoms does not destroy the framework structure of ZIF-8 (Fig. S8) [46]. Furthermore, there is no evident metal-based crystal phase in any of the prepared catalysts (Fig. S9). There is no evidence for metallic nanoparticle formation. The atomic structure was investigated by aberration-corrected high-angle annular dark-field scanning transmission electron microscopy (HAADF-STEM). As shown in Fig. 1C, paired bright spots are randomly distributed on MoFe–N–C, and no metal clusters or particles are observed. The atomic distance was obtained by the intensity profiles from the HAADF-STEM as shown in Fig. 1D. Two typical pairs from the yellow boxes show distances of 2.11 and 2.28 Å. We counted the distances of 100 pairs of atoms and find a value of 2.23 ± 0.32 Å (Fig. 1E). Electron energy loss spectroscopy (EELS) confirms that the paired bright spots correspond to Mo and Fe atoms (Fig. S10). Notably, no significant Zn signal was observed in EELS, due to the sublimation of Zn atoms during high-temperature annealing. The energy-dispersive X-ray spectroscopic (EDS) elemental mapping analyses further show the uniform distribution of C, N, Mo and Fe atom on FeMo–N–C (Fig. 1F–J).

**Fig. 2:**
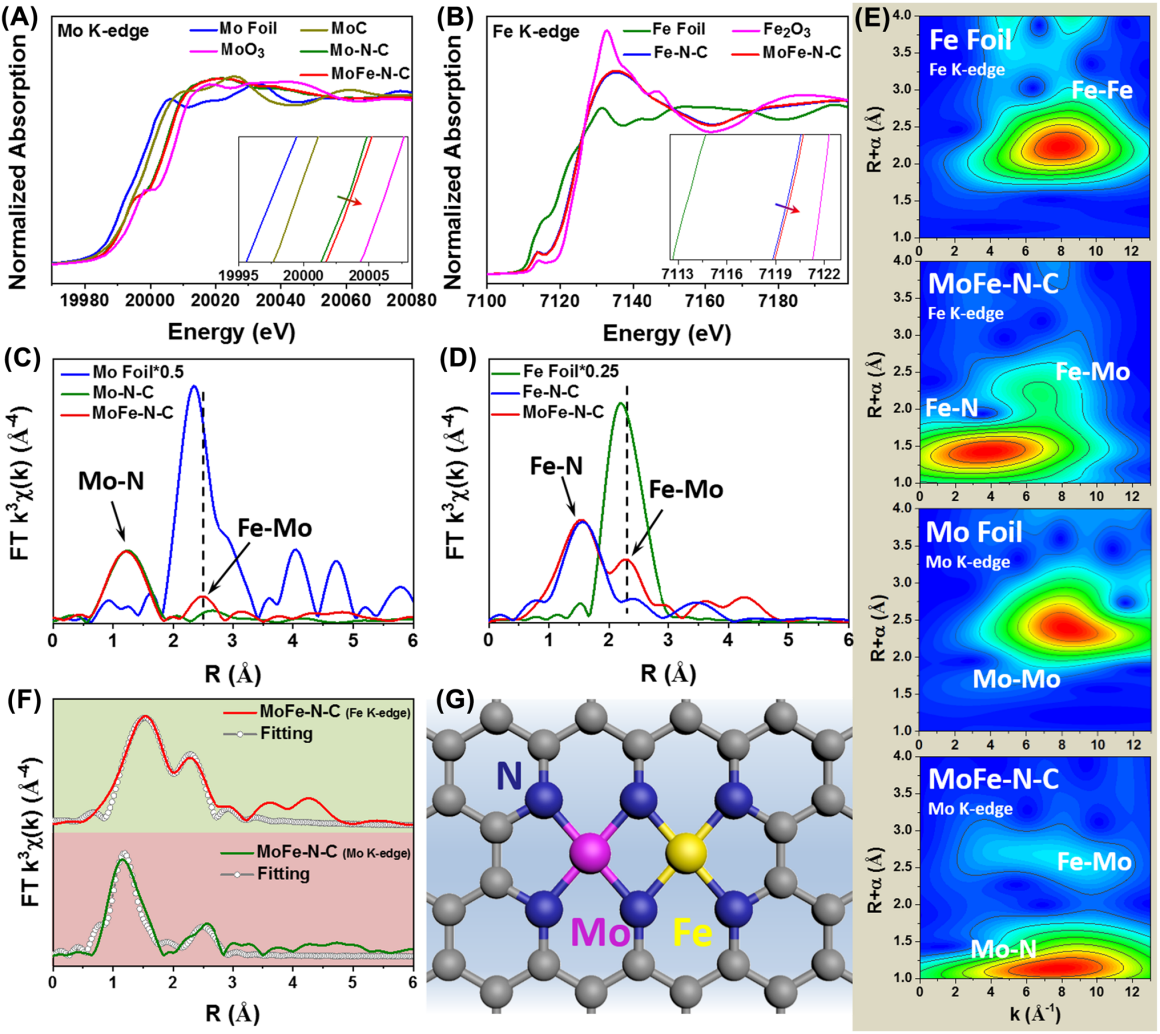
**A** Mo K-edge XANES spectra of the Mo foil, MoC, MoO_3_, Mo–N–C and MoFe–N–C. **B** Fe K-edge XANES spectra of Fe foil, Fe_2_O_3_, Fe–N–C and MoFe–N–C. FT-EXAFS spectra at Mo K-edge (**C**) and Fe K-edge (**D**), respectively. **E** WT-EXAFS spectra of different samples. **F** FT-EXAFS spectra fitting curves of MoFe-N–C. **G** Schematic atomic interface model for MoFe–N–C

The elemental composition and chemical states were investigated by X-ray photoelectron spectroscopy (XPS) and X-ray absorption fine-structure (XAFS). The high-resolution Mo 3*d* spectra and Fe 2*p* spectra confirmed that the metal atoms were successfully anchored on the substrate (Fig. S11). There is no evidence for the presence of metal-based carbides in the C 1*s* spectra for the four samples (Fig. S12 and Table S1), consistent with the above XRD and TEM results [30, 47]. Notably, the peaks for Mo N–C, Fe–N–C and MoFe–N–C samples at 399.4 eV in the N 1*s* spectra are attributed to metal-N (M-N_x_) bonds, and the proportion of M-N_x_ bonds in MoFe–N–C is higher than that of single atoms catalysts (Fig. S13 and Table S2). These evidences suggesting that the transition metal atoms are anchored on the carbon carrier via M–N coordination [26, 48]. The chemical valence was investigated by X-ray absorption near-edge structure (XANES) measurements. The near-edge absorption energy of Mo–N–C and MoFe–N–C in the Mo K-edge XANES spectra (Fig. 2A) is located between MoC and MoO_3_, suggesting that the oxidation state of Mo in the two catalysts is intermediate between Mo^2+^ and Mo^6+^ [49, 50]. Similarly, the Fe element oxidation states of Fe–N–C and MoFe–N–C are between Fe foil (Fe^0^) and Fe_2_O_3_ (Fe^3+^) (Fig. 2B). Furthermore, the near-edge absorption energy of MoFe–N–C in the Mo K-edge and Fe K-edge XANES spectra is positively shifted compared with Mo–N–C and Fe–N–C, respectively. Thus, the oxidation states of Mo and Fe on MoFe–N–C are slightly higher than those on Mo–N–C and Fe–N–C, indicating that the Mo-Fe pair transfers more charge to the coordinated N atom [30, 38].

The corresponding Fourier transformed (FT)-EXAFS spectra were investigated. These show significant differences in the local coordination of the metal sites among those catalysts. As shown in Fig. 2C, the Mo K-edge FT-EXAFS spectra of Mo–N–C and MoFe–N–C shows a main peak at ~ 1.23 Å. This is ascribed to the Mo–N path [51, 52]. Figure 2D shows the Fe K-edge FT-EXAFS spectra of Fe–N–C and MoFe–N–C with a main peak at ~ 1.50 Å attributed to the Fe–N path [53, 54]. Importantly, both FT-EXAFS spectra of MoFe–N–C have a peak assigned to Fe-Mo bond. This is significantly different from Mo-Mo bond and Fe–Fe bond. Importantly, this is further evidence for the existence of Mo-Fe diatomic pairs [53, 54]. Wavelet transforms (WT)-EXAFS were also done to identify the metal-N and metal–metal paths (Fig. 2E). The Fe K-edge WT-EXAFS spectra of MoFe-N–C show intensity maxima at ~ 3.8 Å^−1^ corresponding to Fe–N path, while the Mo K-edge WT-EXAFS spectra of MoFe-N–C show an intensity maxima at ~ 7.4 Å^−1^ assigned to Mo–N path. Both the Mo and Fe K-edge WT-EXAFS spectra of MoFe-N–C have a signal located at ~ 7.1 Å^−1^ corresponding to Mo-Fe paths. This is significantly different from Mo-Mo bonds (~ 8.3 Å^−1^) and Fe–Fe bonds (~ 8.0 Å^−1^). The WT-EXAFS signals are consistent with the FT-EXAFS results, confirming the presence of M–N coordination and Mo-Fe bonds in MoFe–N–C. Furthermore, the specific coordination configuration of metal sites was surveyed by quantitative least-squares EXAFS curve fittings to investigate the structure parameters. The best-fit result indicates that the coordination number (C.N.) for Mo–N and Fe–N bonding in the MoFe-N–C catalyst both is approximately 4 with atomic distances of 1.68 ± 0.04 and 1.97 ± 0.02 Å, respectively (Figs. 2F, S14 and Table S3). There is also a Mo-Fe path with a C.N. of ~ 1 and a bond length of ~ 2.35 Å, which is significantly different from the Mo-Mo bond (2.72 ± 0.01 Å) and the Fe–Fe bond (2.45 ± 0.01 Å) (Fig. S15). Again this supports the formation of Mo-Fe pairs rather than metal-based nanoparticles. Figure 2G shows the structural model of MoFe–N–C used for EXAFS curve fitting, which is in agreement with experimental spectra. Therefore, the MoFe-N_6_ model consisting of a Mo-Fe pair with six coordination N atoms is suggested as the probable structure for MoFe-N–C, with the local coordination structure for Mo–N–C and Fe–N–C of Mo-N_4_ and Fe-N_4_, respectively (Fig. S16 and Table S3).

### Electrocatalytic Performance

**Fig. 3:**
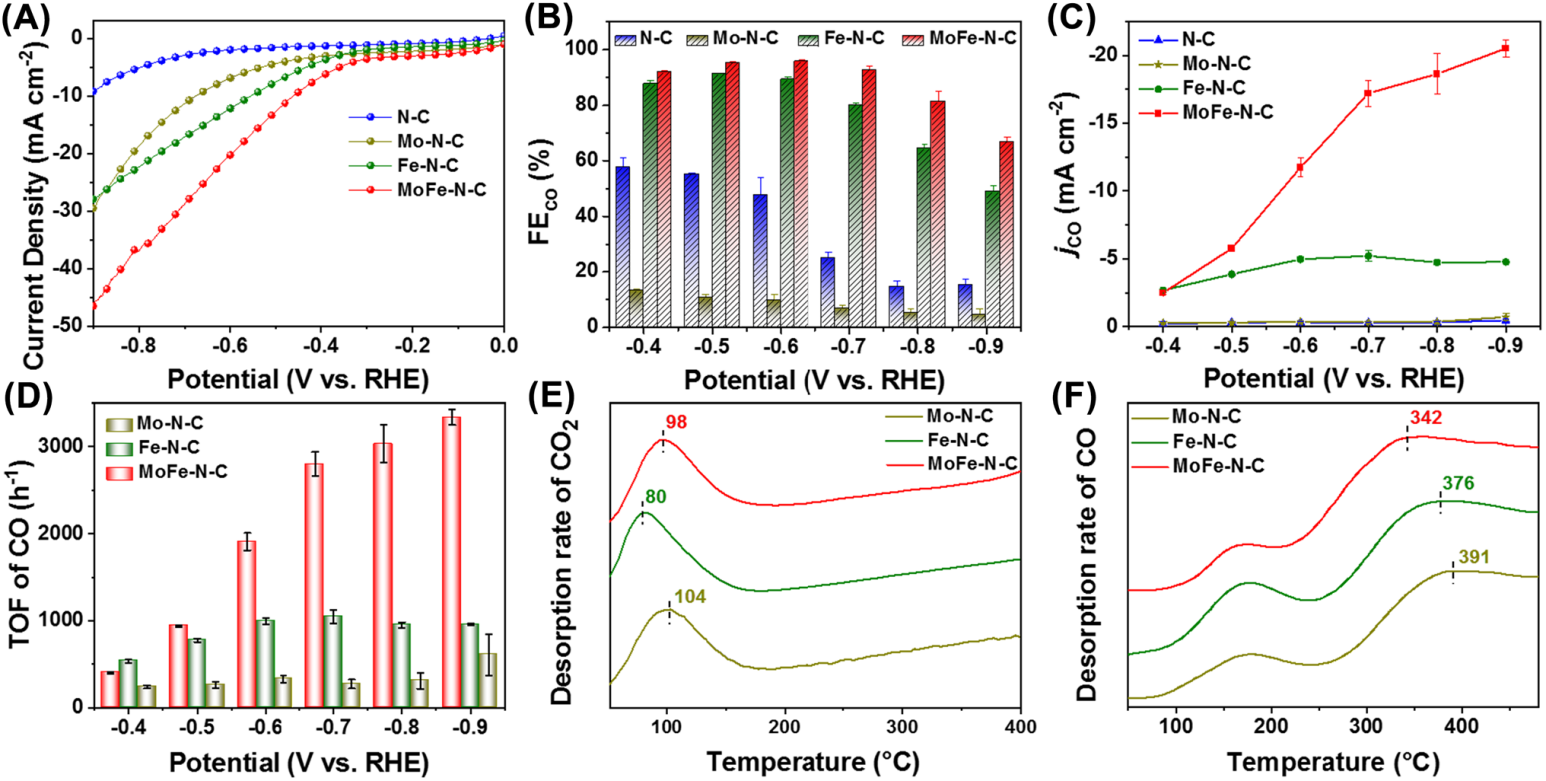
Electrocatalytic Performance in the flow cell: **A** LSV curves of N–C, Mo–N–C, Fe–N–C and MoFe–N–C measured in CO_2_-saturated 0.5 M KHCO_3_ electrolyte. **B** CO Faradaic efficiency and **C** CO partial current densities of the different catalysts at selected potentials. **D** TOFs for CO production of Mo–N–C, Fe–N–C and MoFe–N–C. **E** CO_2_ and **F** CO temperature-programmed desorption of the Mo–N–C, Fe–N–C and MoFe–N–C

The electrochemical activities of the catalysts for CO_2_RR were evaluated by linear sweep voltammetry (LSV). The results are shown in Fig. 3A. MoFe–N–C presents a higher current density as compared with N–C, Mo–N–C and Fe–N–C catalysts, suggesting that MoFe–N–C has higher electrochemical activity. The occurrence of CO_2_RR was confirmed by a control experiment, specifically the fact that MoFe–N–C shows higher current density in CO_2_-saturated electrolyte than in Ar-saturated electrolyte (Fig. S17). The catalytic activities of MoFe–N–C with metal contents were investigated (details in Supporting Information, Figs. S18–S25, Tables S4 and S5). The catalytic activity and selectivity were increased at first and then decreased with the increase of Mo content and Fe content. This is because the initial increase of Mo and Fe improves the density of Mo-Fe pairs. But the number of unpaired Mo sites increased with the further raise of Mo content, which leads to the enhancement of competitive HER. In addition, excess Fe causes the agglomeration of metal atoms, which degrades its catalytic activity and selectivity. The optimal contents of Mo and Fe on MoFe–N–C are 0.21 and 0.52 wt%, respectively.

Potentiostatic electrolysis was conducted to evaluate the reduction products in a flow cell. Only CO and H_2_ were detected in the CO_2_RR products by on-line gas chromatography (GC) and hydrogen nuclear magnetic resonance (^1^H NMR) spectroscopy (Fig. S26). Meanwhile, MoFe-N–C reaches a maximum FE_CO_ of 95.96% at − 0.60 V. This is higher than that of N–C (47.73%), Mo–N–C (9.82%) and Fe–N–C (89.39%) (Figs. 3B and S27). Furthermore, the MoFe-N–C exhibited a FE_CO_ above 90% over a broad potential window of − 0.40 to − 0.70 V. CO partial current density (*j*_CO_) was calculated based on the FE_CO_ at different potentials (Fig. 3C). The *j*_CO_ of MoFe–N–C was up to − 11.72 ± 0.70 mA cm^−2^ at − 0.60 V is higher than that of Fe–N–C (− 4.97 ± 0.17 mA cm^−2^), Mo–N–C (− 0.38 ± 0.05 mA cm^−2^) and N–C (− 0.33 ± 0.05 mA cm^−2^) at the same applied potential. These results indicate that MoFe–N–C significantly improves the electrocatalytic activity for CO_2_RR compared with N–C, Mo–N–C and Fe–N–C. The intrinsic activities of the catalysts were further quantified by the turnover frequency (TOF) based on the metal sites (Fig. 3D). The MoFe–N–C attained a high TOF value of 3336.62 h^−1^ at − 0.90 V. This is 3.5-fold higher as compared to Fe–N–C (957.44 h^−1^) and approximately 5.5-fold higher than Mo–N–C (610.74 h^−1^). The FE and TOF values for MoFe-N–C are superior to those of previously reported Fe-based SACs (Table S6). The above results show the outstanding intrinsic activity and selectivity of the MoFe–N–C catalyst for electrochemical CO_2_RR.

The stability was investigated by chronoamperometry measurements of the MoFe–N–C (at − 0.60 V for 100 h) (Fig. S28). The MoFe–N–C catalyst retained over 96.83% of its initial CO selectivity, without apparent changes in the total current density during continuous operation. Importantly, there are no obvious differences for physicochemical properties of morphology, size and crystallinity of the MoFe–N–C before and after potentiostatic electrolysis (Fig. S29A, B). No obvious bulk-like metallic phases are observed from XRD patterns and HR-TEM images. EDS mapping shows the homogeneous distribution of C, N, Mo and Fe over the whole nanostructure (Fig. S29C–G). All these evidences show that the metal atoms are still uniformly dispersed on MoFe–N–C after potentiostatic testing, demonstrating the outstanding structural stability of MoFe–N–C.

CO_2_ and CO temperature-programmed desorption (TPD) measurements were carried out in order to understand the performance. As shown in Fig. 3E, all catalysts exhibit a TPD signal peak combined by physisorption peak and chemisorption peak from 50 to 150 °C [19, 55]. MoFe–N–C and Mo–N–C catalysts displayed higher CO_2_ desorption temperature than Fe–N–C, indicating stronger CO_2_ adsorption. Figure 3F shows the CO TPD spectrums of the Mo–N–C, Fe–N–C and MoFe–N–C. The low temperature peaks at around 150 °C are ascribed to the physisorption associated with van der Waals forces, while peaks at about 350 °C can be ascribed to chemisorption associated with weak chemical bonds [56]. We focused on CO chemisorption, which may be associated with the electrochemical adsorption of *CO intermediates at the active site [57]. The results show that the chemisorption peak of MoFe–N–C is located at 342 °C, lower than other samples. Thus, CO adsorbed on the MoFe–N–C more readily desorbs as compared to the other materials. This is consistent with its CO_2_RR catalytic activity. Although the single atom Mo–N–C has the strongest CO_2_ adsorption capacity, CO desorption on that material is difficult, which weakens its catalytic activity. In contrast, the higher CO_2_RR catalytic activity of MoFe–N–C is derived from its stronger CO_2_ adsorption capacity and easier CO desorption. Thus, MoFe–N–C breaks the linear scaling relationship, and this leads to high catalytic performance.

### DFT Calculations

**Fig. 4:**
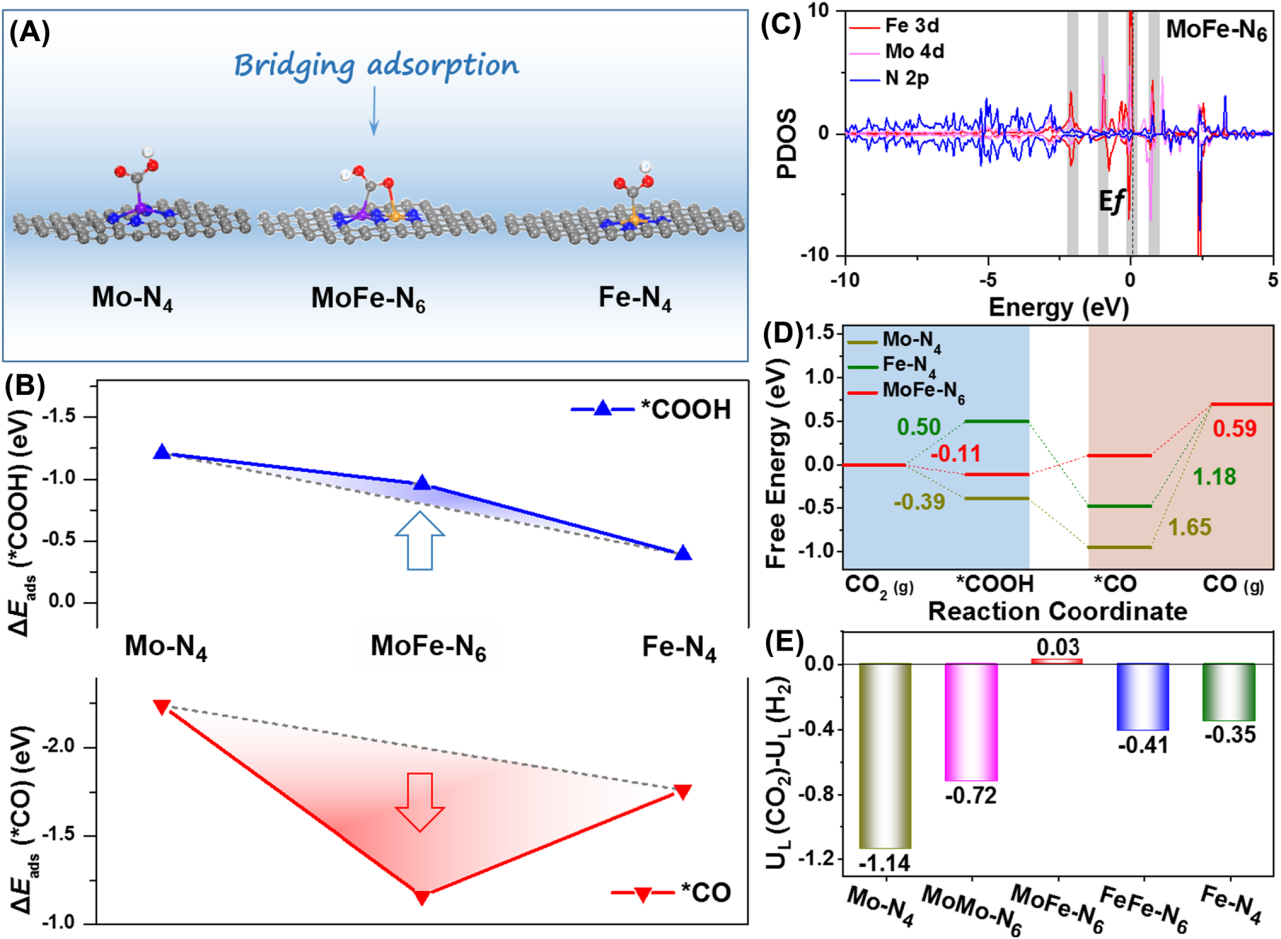
**A** Optimized catalytic models and reaction pathways on MoFe-N_6_. Purple, yellow, blue, red, white and gray represent the Mo, Fe, N, O, H and C atoms, respectively. **B** Absorption energy of *COOH intermediates and *CO intermediates on different catalytic sites. **C** Calculated PDOS of MoFe-N_6_. **D** Free energy diagrams of the catalysts for CO_2_RR. **E** Difference in the limiting potentials for CO_2_RR and HER

DFT calculations were carried out to understand how the linear scaling relationship is broken leading to the intrinsic activity of MoFe–N–C for CO_2_RR. To further explore the reasonable existence of the MoFe–N_6_ structure, we constructed six typical structures (Fig. S30). As mentioned above, we captured Fe atoms on the basis of Mo N–C with stable Mo-N_4_ structure, thus excluding homologous bimetallic sites. The optimized geometries and their corresponding total energies (Table S7) show that the MoFe-N_6_ structure has the lowest total energy. The lower the total energy of the system, the more stable the structure, so MoFe-N_6_ is the most reasonable structure. The distance between the Mo and Fe atoms in the optimized MoFe-N_6_ model is ~ 2.31 Å. This is close to the value obtained from the HAADF-STEM image in Fig. 1C, supporting the model. Structural models of Mo-N_4_, Fe-N_4_, and MoFe-N_6_ during CO_2_RR process were also constructed on the basis of the EXAFS fitting results (Fig. S31). Figure 4A shows the adsorption of *COOH intermediates on Mo-N_4_, Fe-N_4_ and MoFe-N_6_ during CO_2_RR process. We found that the C and an O atom of *COOH simultaneously interact with the Mo and Fe atoms of MoFe-N_6_ sites. This is a key difference from the other materials, which lack hetero-diatomic pairs. In particular, this bridging adsorption pattern was not observed on Mo-N_4_ and Fe-N_4_ sites. We also compared the catalytic paths of *COOH intermediate adsorbed alone on Mo or Fe atoms in the MoFe-N_6_ structure (Fig. S32). The results show that *COOH intermediate requires the lowest energy to be adsorbed on Mo-Fe atom pairs by bridge adsorption. At the same time, it is more conducive to the stability of *COOH intermediate and the subsequent reaction. The charge density difference (Fig. S33) and PDOS (Fig. S34) of the catalytic sites adsorbed with *COOH intermediate indicate that this bridging adsorption improves the interaction between *COOH intermediate and MoFe-N_6_ site by simultaneously transferring electrons from Mo and Fe atoms to *COOH intermediate. The adsorption energy of *COOH intermediate and *CO intermediate at different catalytic sites is shown in Fig. 4B. Compared with the Fe-N_4_ site, MoFe-N_6_ site presented higher adsorption energy for *COOH intermediate due to bridge adsorption. However, the *CO intermediate adsorption energy on the MoFe-N_6_ site is smaller than that of other catalyst models. Thus, the limitation of the linear scaling relationship is broken. The PDOS are presented in Figs. 4C and S35, and the charge distributions are in Fig. S36. The calculated PDOS shows that the Mo 4*d* and Fe 3*d* orbitals of MoFe-N_6_ site mix strongly near the Fermi level, implying a strong d-d orbital coupling in the Mo-Fe hetero-diatomic pair [26]. In addition, electron delocalization was observed on MoFe-N_6_ site, and in particular more electrons of the metal site transfer to the coordinating N atoms. Related to this, the electron transfer between the *CO intermediate and the MoFe-N_6_ sites is reduced. This reduced electron transfer may then be expected to reduce the adsorption energy of *CO intermediates and facilitate *CO desorption (Figs. S37 and S38) [19], as in fact is found. Figure 4D shows the free energy diagrams of the catalysts for CO_2_RR. The MoFe-N_6_ site not only has the lowest *CO desorption energy, it also provides spontaneous formation of *COOH intermediates. These results show that the MoFe-N_6_ site successfully breaks the limitation of linear scaling relationships through bridging adsorption and d-d orbital coupling.

Theoretical models for MoMo-N_6_ and FeFe-N_6_ sites were constructed to further test the role of bridging adsorption and orbital coupling in regulating the ad-desorption energy of intermediates (Fig. S39). It was found that MoMo-N_6_ site also provides bridging adsorb *COOH intermediates and improves the adsorption energy of *COOH intermediates (Fig. S40). However, its larger adsorption energy of *CO intermediates would limit the *CO desorption (Fig. S41). On the other hand, FeFe-N_6_ site shows the electron delocalization that facilitates *CO intermediate desorption. However, it also reduces the adsorption energy of *COOH intermediates (Fig. S42). Thus, the hetero-diatomic pairs are crucial for the performance. In particular, the orbital coupling between Mo and Fe atoms facilitates *CO intermediates desorption, while the bridging adsorption of *COOH by Mo-Fe pairs significantly reduces the formation energy of *COOH intermediates.

Finally, we note that HER is the main competing reaction for CO_2_RR (Fig. S43). In this regard, the limiting potential difference between CO_2_RR and HER (*i.e.*, Δ*U* = *U*_L_(CO_2_)—*U*_L_(H_2_), where *U*_L_ = − Δ*G*_0_/*e*) was calculated as a measure of CO_2_RR selectivity. As shown in Fig. 4E, the MoFe-N_6_ site shows more positive Δ*U* value, indicating its higher CO_2_RR selectivity. These findings suggest that MoFe-N_6_ sites more outstanding excellent activity and selectivity toward CO_2_RR-to-CO, in accord with our experimental results.

## Conclusions

In summary, we have developed a hetero-diatomic catalyst based on dispersed Mo-Fe hetero-pairs anchored on a N-doped carbon matrix. This breaks the linear scaling relationship leading to high electrocatalytic CO_2_RR performance. The prepared MoFe–N–C catalyst exhibits high CO_2_RR intrinsic activity (reaching a maximum TOF of 3336 h^−1^), high selectivity (displayed a FE_CO_ of 95.96% at − 0.6 V) and excellent stability. Density functional theory calculations show that the bridging adsorption of *COOH intermediates by hetero-diatomic sites reduces the formation energy of *COOH. Simultaneously, the d-d orbital coupling between the Mo-Fe pair results in electron delocalization and facilitates *CO desorption. This work provides an important direction for the rational design of hetero-diatomic catalysts for targeted catalysis and provides an in-depth understanding of the structure–activity relationships of these systems at the atomic scale.

## Supplementary Information

Below is the link to the electronic supplementary material.

Supplementary file1 (PDF 4183 KB)
