# Supplementary material for: Atomic Dispersed Hetero-Pairs for Enhanced Electrocatalytic CO2 Reduction
Source: Nanomicro Lett. 2023 Nov 6;16:4. doi: 10.1007/s40820-023-01214-2 (PMC10628116; doi:10.1007/s40820-023-01214-2)
Supplement: Supplementary file 1 — Supplementary file1 (PDF 4183 KB) [file 40820_2023_1214_MOESM1_ESM.pdf]

Supporting Information

Atomic Dispersed Hetero-Pairs for Enhanced Electrocatalytic CO_2 Reduction

Zhaoyong Jin[#, 1], Meiqi Yang[#, 2], Yilong Dong[1], Xingcheng Ma[1], Ying Wang[1], Jiandong Wu[1], Jinchang Fan[1], Dewen Wang[1], Rongshen Xi[1], Xiao Zhao[1], Tianyi Xu[1], Jingxiang Zhao[2, *], Lei Zhang[3, *], David J. Singh[4], Weitao Zheng[1, *] and Xiaoqiang Cui[1, *]

[1] State Key Laboratory of Automotive Simulation and Control, School of Materials Science and Engineering, Key Laboratory of Automobile Materials of MOE, Electron Microscopy Center, Jilin University, Changchun 130012, P. R. China

[2] Key Laboratory of Photonic and Electronic Bandgap Materials, Ministry of Education, and College of Chemistry and Chemical Engineering, Harbin Normal University, Harbin 150025, P. R. China

[3] College of Chemistry, Jilin University, Changchun 130012, P. R. China

[4] Department of Physics and Astronomy and Department of Chemistry, University of Missouri, Columbia, Missouri 65211, United States

*Corresponding authors. E-mail: zhaojingxiang@hrbnu.edu.cn (Jingxiang Zhao);, lzhang@jlu.edu.cn (Lei Zhang); wtzheng@jlu.edu.cn (Weitao Zheng); xqcui@jlu.edu.cn (Xiaoqiang Cui)

S1 Experimental Section

S1.1 Characterization

The morphologies were obtained with an SU8010 field emission scanning electron microscopy (FESEM) at an accelerating voltage of 5 kV (Hitachi, Japan). The structure of the prepared samples was characterized by powder X-ray diffraction (XRD) on a Bragg-Brentano diffractometer equipped with a Cu-Kα (λ = 0.15418 nm) emitting source (D8-tools, Germany). The scanning speed was set to 5° min^{-1}. Transmission electron microscope (TEM), high-resolution transmission electron microscope (HRTEM) was acquired by using a JEM-2100F transmission electron microscope (JEOL Co., Japan). High angle annular dark field (HAADF) images, electronic energy loss spectroscopy (EELS) and energy-dispersive X-ray (EDX) mapping were obtained on a JEM-ARM300F GRAND ARM transmission electron microscope operating at 300 kV (JEOL Co., Japan). Inductively coupled plasma optical emission spectroscopy (ICP-OES) data was collected with an iCAP7600 ICP-OES system (Thermo Fisher Scientific, USA). X-ray photoelectron spectroscopy (XPS) results were acquired with an ESCALAB-250 instrument, examined with a monochromatic Al-Kα (1486.6 eV) radiation source and a hemisphere detector with

an energy resolution of 0.1 eV (Thermo Fisher Scientific, USA). Fe K-edge and Mo K-edge X-ray absorption spectra was collected at Beijing Synchrotron Facility (BSRF) on beamline 1W1B (XAFS station) at 2.2 GeV. Fe foil, Mo foil, Fe_2O_3, MoC and MoO_3 powder were used as references. All the data were collected at ambient temperature applied in the transmission mode. The EXAFS raw data were processed using the ATHENA module implemented in the IFEFFIT software packages S5 according to the standard procedures of the background-subtracted, normalized and Fourier transformed. For the EXAFS fitting, theoretical scattering paths (Mo-Mo, Fe-Fe, Fe-Mo, Mo-N and Fe-N) were calculated with FEFF6 using Artemis. The obtained XAFS data was processed in Athena (version 0.9.26) for background, pre-edge line and post-edge line calibrations. Then Fourier transformed fitting was carried out in Artemis (version 0.9.26). All EXAFS spectra were fit to the distance (ΔR), CN, and mean-square displacement of interatomic distance (σ^2) using the Artemis interface with a fixed amplitude reduction factor (S_0^2) of 0.55 or 0.98. The parameters such as interatomic distance (R), coordination number (CN), the difference in threshold energy (ΔE_0) and Debye-Waller factor (σ^2) were first established with reasonable estimation and then were fitted in the R domain. The error in the overall fits was determined using the *R*-factor, the goodness-of-fit parameter, in which R-factor = $\Sigma(\chi_{data}-\chi_{fit})^2/\Sigma(\chi_{data})^2$ and good fits occurred for an R-factor < 0.05. For Wavelet Transform analysis, the χ(k) exported from Athena was imported into the Hama Fortran code. The parameters were listed as follow: *R* range is 1-4 Å, k range is 0 - 10 $Å^{-1}$; *k* weight is 3; and Morlet function with κ=10, σ=1 was used as the mother wavelet to provide the overall distribution. N_2 adsorption-desorption isotherms, surface areas, and pore size distributions were recorded at 77 K on an ASAP 2420 volumetric gas sorption instrument (Micromeritics, USA). Temperature-programmed desorption of CO_2 (CO_2-TPD) and CO (CO-TPD) were performed using an Autochem II 2920 analyzer (Micromeritics, USA).

S1.2 Product Analysis

S1.2.1 Gas Product Analysis

CO_2 reduction products were measured *via* gas chromatography (GC, Shimadzu, GC-2014) and nuclear magnetic resonance (NMR, Bruker 600 MHz) for the gas and liquid products, respectively. High purity helium (99.999%) was used as the carrier gas. The faradaic efficiencies (FE) of the CO or H_2 were calculated by using the concentrations (ppm) detected by the GC as follows:

$$FE=\frac{Q_g}{Q_{total}}=\frac{x_g \times V_{CO2} \times n \times F}{\int_0^t I dt}\times 100\% \qquad (S1)$$

Where x_g is the concentration of gas production measured by GC (mol/mol), V_{CO2} is the flow rate (mol s^{-1}) of high-purity CO_2, controlled by a mass flow controller, *n* is the number of electrons transferred for the product formation, *F* is the Faraday constant (96,485 C mol^{-1}), *I* is the total current density of the chronoamperometry curve.

The turnover frequency (TOF, h^{-1}) of the Mo-N-C ($TOF_{Mo\text{-}N\text{-}C}$), Fe-N-C($TOF_{Fe\text{-}N\text{-}C}$) and MoFe-N-C($TOF_{MoFe\text{-}N\text{-}C}$) were calculated based on the following formula:

$$TOF_{\text{Mo-N-C}} = \frac{I_{product} / NF}{m_{cat.} \times w_{Mo} / M_{Mo}} \times 3600 \qquad (S2)$$

$$TOF_{Fe\text{-}N\text{-}C} = \frac{I_{product} / NF}{m_{cat.} \times w_{Fe} / M_{Fe}} \times 3600 \qquad (S3)$$

$$TOF_{MoFe\text{-}N\text{-}C} = \frac{I_{product} / NF}{m_{cat.} \times w_{Mo} / M_{Mo} + m_{cat.} \times w_{Fe} / M_{Fe}} \times 3600 \qquad (S4)$$

Where $I_{product}$ is the partial current for CO; N is the number of electrons required to produce a CO molecule, which is 2; F is Faradaic constant (96485 C/mol); $m_{cat.}$ is the mass of the catalyst deposited on the carbon paper electrode; w_{Mo} and w_{Fe} are the mass fractions of Mo and Fe based on ICP-AES results, respectively; M_{Mo} and M_{Fe} are the atomic mass of Mo and Fe, respectively.

S1.2.2 ^{1}H NMR Measurements and Analysis Details

The liquid products were detected by off-line 600 MHz ^{1}H nuclear magnetic resonance measurements (NMR, AVANCE III 600, Bruker). The ^{1}H NMR spectrum was measured with water suppression *via* a presaturation method. To quantify the liquid products, 0.3 mL fresh electrolyte electrolyzed at a determined potential for 2 h was mixed with 0.28 mL D_2O and 0.02 mL DMSO. The ratio of the peak areas of the obtained liquid products to the DMSO peak area were compared to quantify the concentrations of the reaction products. Then, the Faradaic efficiency of liquid products (FE_{liquid}) was calculated as follows:

$$FE_{liquid}(\%) = \frac{n \times V \times F \times N_k}{Q} \times 100\% \qquad (S5)$$

Where n (mol) is the content of liquid products, based on the calibration of the NMR; V is the electrolyte volume in the cathodic chamber; F is the Faradaic constant (96485 C mol^{-1}); N_k is the number of transferred to produce species k; Q is the total charge amount at different applied potentials.

S1.3 Computational Details

DFT calculations were performed by the Vienna Ab *Initio* Simulation Package (VASP) with the projector augmented-wave method [1, 2]. We considered electronic exchange and correlation using the Perdew-Burke-Ernzerhof (PBE) functional, employing the generalized gradient approximation (GGA) [3]. All these computations were reported using a plane-wave energy cutoff of 500 eV. The convergence criteria for the forces and energies were 0.05 eV Å^{-1} and 10^{-5} eV, respectively. Van der Waals interactions were included in Grimme's empirical (DFT + D3) method [4]. The Brillouin zone was sampled using k-point on a 3 × 3 × 1 Monkhorst-Pack grid [5]. The denser k-point (9 × 9 × 1) was utilized to calculate the electrical structure. We

constructed simulated models for metal-nitrogen-doped carbon catalysts using graphene supercell (12.34 Å × 12.83 Å, 60 C atoms) with a periodic boundary. The vacuum space in the z-direction was adjusted to 15 Å to avoid virtual interaction between periodic graphene layers. The adsorption energy (E_{ads}) of the adsorbate on the surface was defined as $E_{ads} = E_{total} - E_{adsorbate} - E_{surface}$, where E_{total}, $E_{adsorbate}$, and $E_{surface}$ are the total energies of the adsorbed species on the surface, isolated adsorbate, and pristine surface, respectively. The computational hydrogen electrode technique proposed by Nørskov et al. was applied to acquire the Gibbs free energy changes (ΔG) of the electrochemical steps of CO_2RR [6, 7]. The Gibbs free energies can be calculated by the following equation:

$$\Delta G = \Delta E + \Delta ZPE - T\Delta S + \int C_p dT - \Delta G_U \qquad (S6)$$

The turnover frequency (TOF, h^{-1}) of the samples was calculated based on the following formula: where ΔE is the reaction energy, ΔZPE is the difference in the zero-point energy between reactants and products, $T\Delta S$ and $\int C_p dT$ are the contributions of entropic and enthalpic energy at room temperature (T = 298.15 K). The effects of applied electrode potential (U) on the free energy of the electron transfer steps; $\Delta G_U = -eU$.

S2 Supplementary Figures and Tables

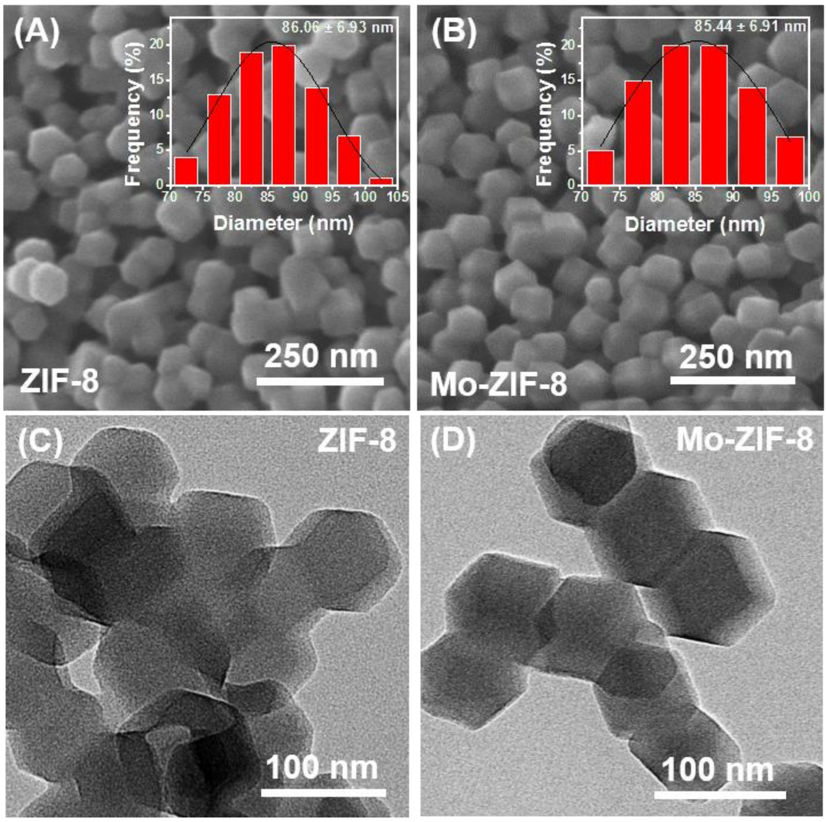

Fig. S1 SEM images of (**A**) ZIF-8, (**B**) Mo-ZIF-8. TEM images of (**C**) ZIF-8, (**D**) Mo-ZIF-8

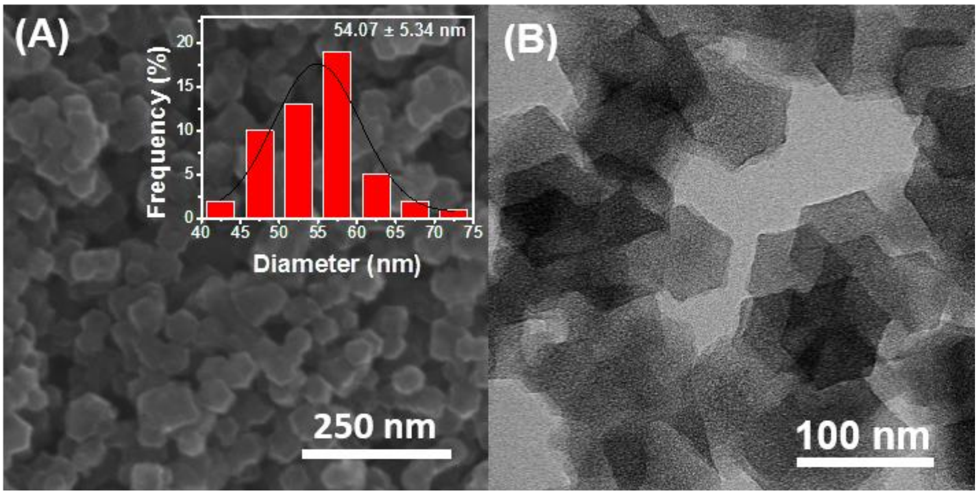

Fig. S2 (**A**) SEM image and (**B**) TEM image of Mo_{first}-N-C

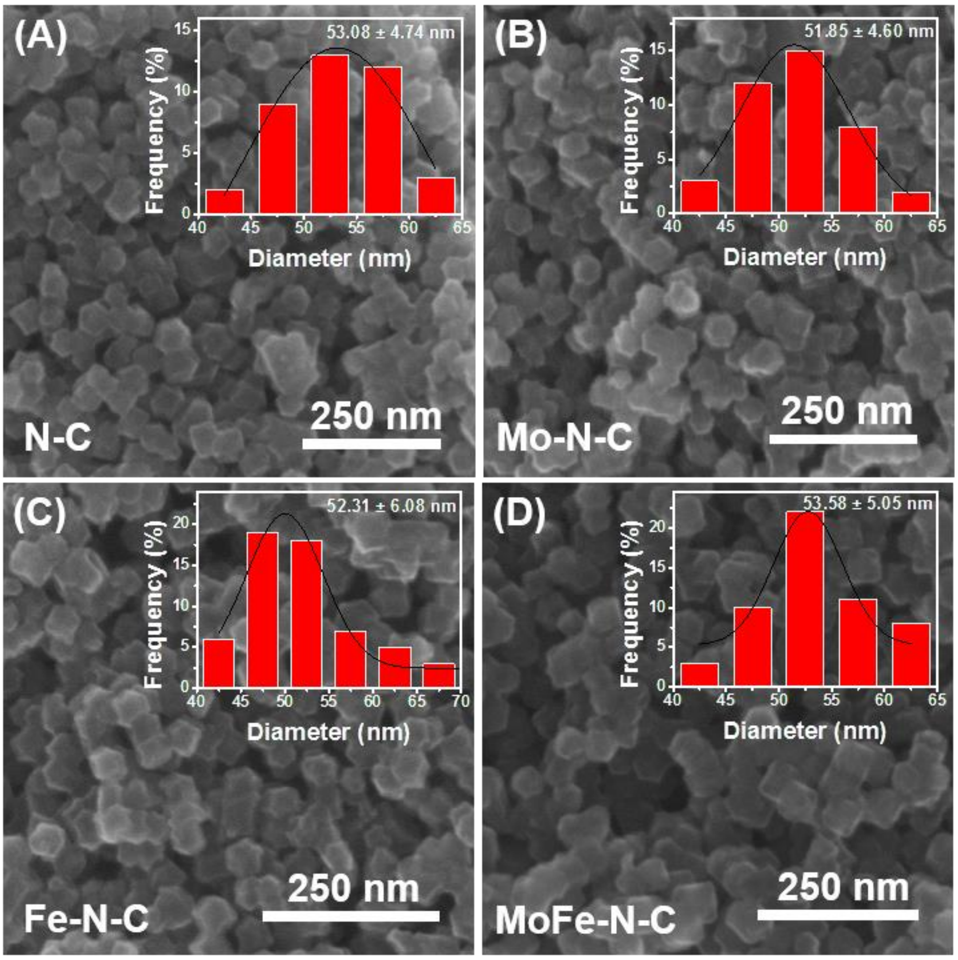

Fig. S3 SEM images of (**A**) N-C, (**B**) Mo-N-C, (**C**) Fe-N-C and (**D**) MoFe-N-C

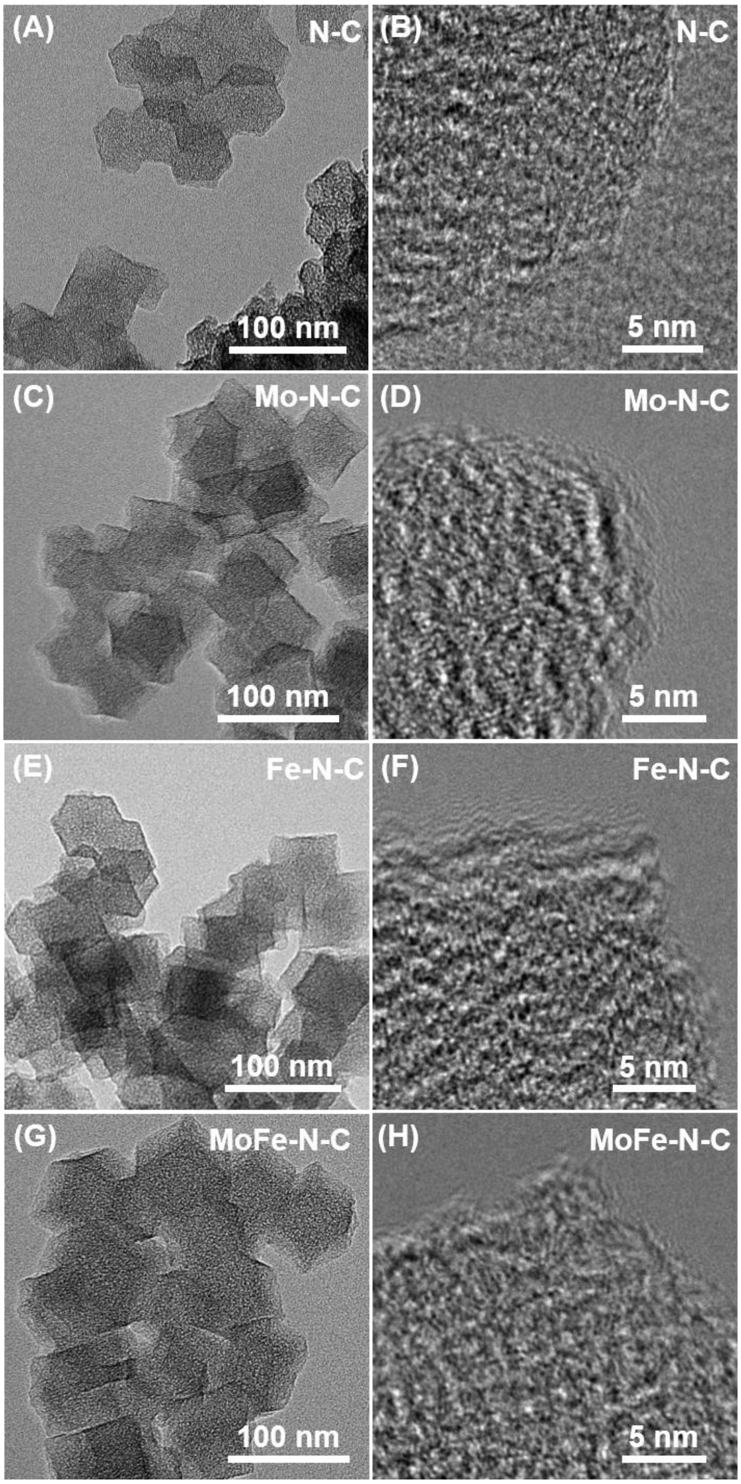

Fig. S4 (**A**) TEM and (**B**) HR-TEM images of N-C, (**C**) TEM and (**D**) HR-TEM images of Mo-N-C, (**E**) TEM and (**F**) HR-TEM images of Fe-N-C, (**G**) TEM and (**H**) HR-TEM images of MoFe-N-C

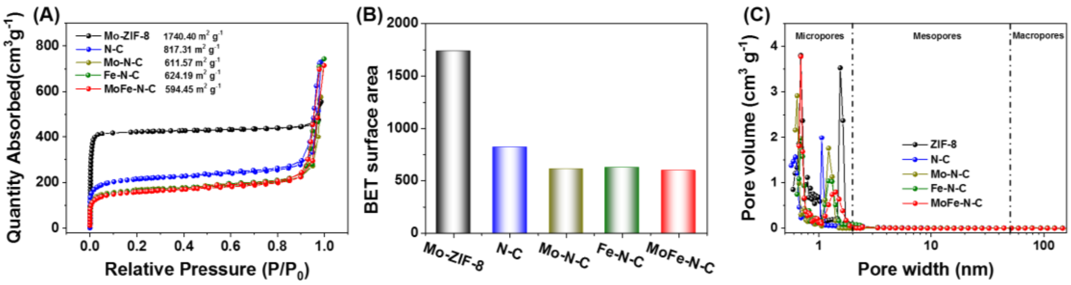

Fig. S5 N_2 adsorption/desorption plots (**A**), BET surface areas (**B**) and pore size distribution (**C**) of Mo-ZIF-8, N-C, Mo-N-C, Fe-N-C and MoFe-N-C

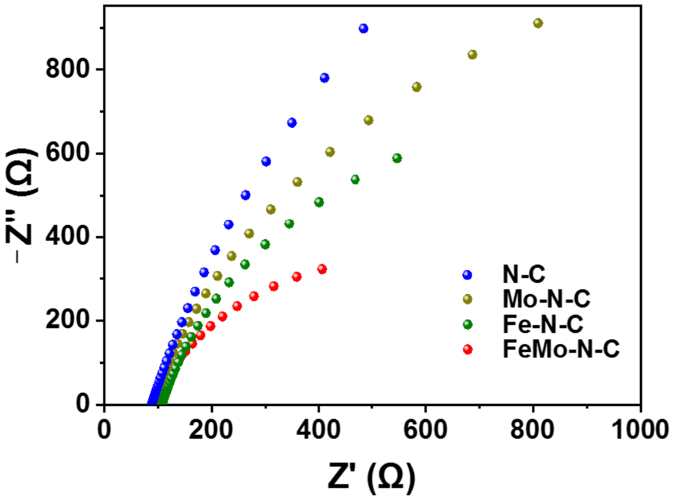

Fig. S6 Electrochemical impedance of N-C, Mo-N-C, Fe-N-C and FeMo-N-C in 0.1 M $KHCO_3$ electrolyte.

Electrochemical impedance results indicate that MoFe-N-C exhibits smaller resistance and faster electron kinetics than N-C, Mo-N-C and Fe-N-C.

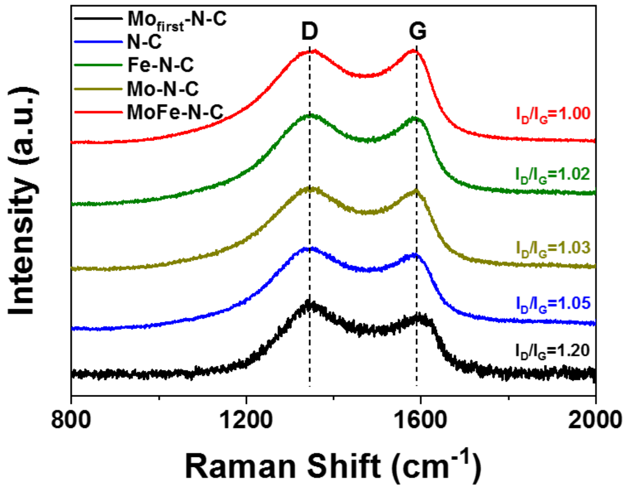

Fig. S7 Raman spectra of Mo_{first}-N-C, N-C, Mo-N-C, Fe-N-C and FeMo-N-C

The Raman spectra show two carbon signal peaks at 1380 cm^{-1} and 595 cm^{-1}, assigned to the D band and G band, respectively. The relative I_D/I_G ratio can be used to evaluate the degree of carbon disorder in carbon nanomaterials. The I_D/I_G ratio of the catalysts were in the following order: Mo$_{first}$-N-C (1.20) > N-C (1.05) > Mo-N-C (1.03) > Fe-N-C (1.02) > MoFe-N-C (1.00). This indicates that the carbon defects are gradually repaired with the increase of annealing times and metal content [8].

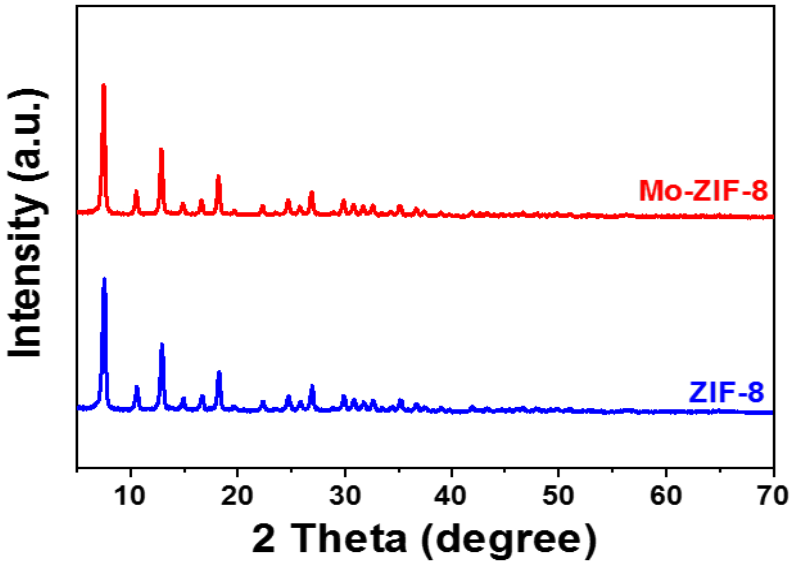

Fig. S8 XRD patterns of the ZIF-8 and Mo-ZIF-8

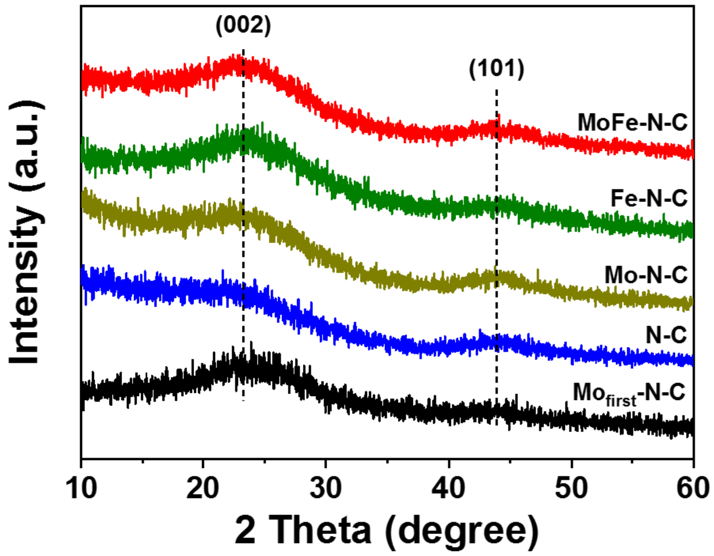

Fig. S9 XRD patterns of Mo$_{first}$-N-C, N-C, Mo-N-C, Fe-N-C and MoFe-N-C.

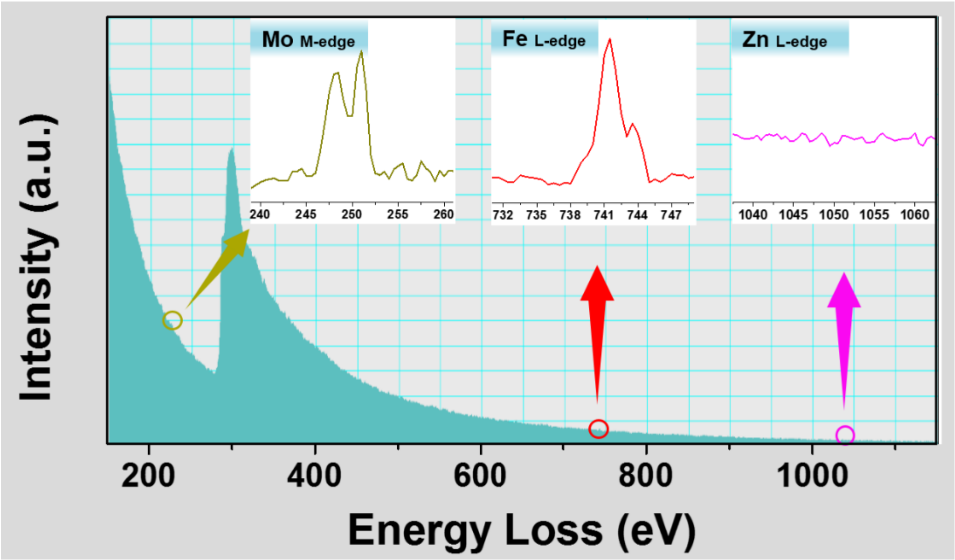

Fig. S10 MoFe-N-C structure analyzed by EELS

Electron energy loss spectroscopy (EELS) of the paired bright spots is show in Fig. S10. Mo-$M_{4,5}$ edges and Fe-$L_{2,3}$ edges were observed at ~250 eV and ~740 eV in one acquisition [9, 10], respectively, which indicates that the paired bright spots corresponding to Fe and Mo atom. Notably, no significant Zn signal was observed.

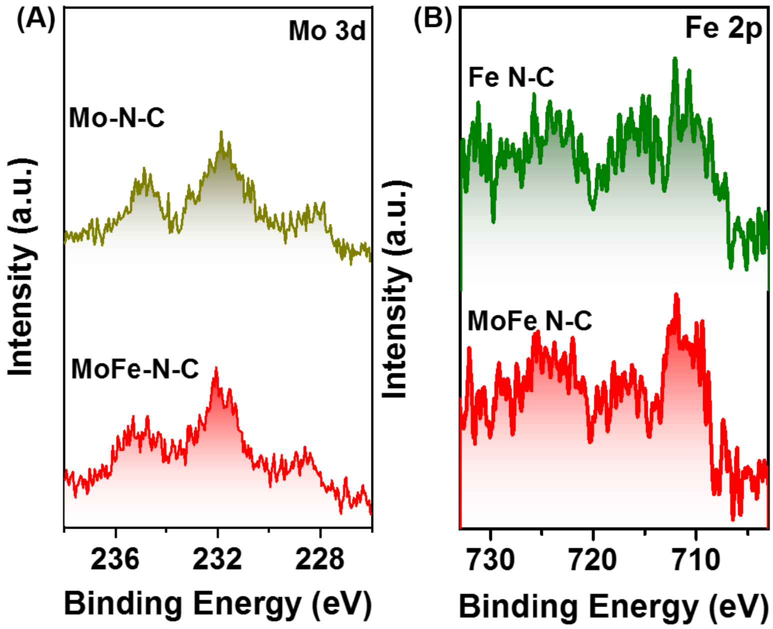

Fig. S11 (**A**) High-resolution Mo 3d spectrums of Mo-N-C and MoFe-N-C. (**B**) High-resolution Fe 2p spectrum of Fe-N-C and MoFe-N-C.

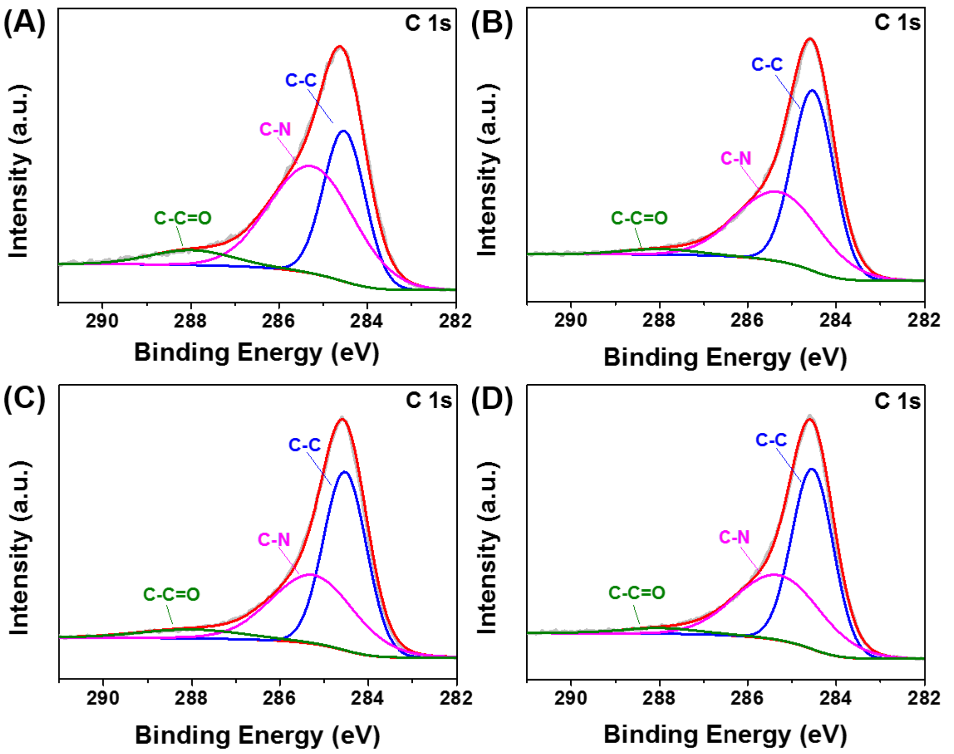

Fig. S12 XPS spectra of C 1s for (**A**) N-C, (**B**) Mo-N-C, (**C**) Fe-N-C and (**D**) MoFe-N-C

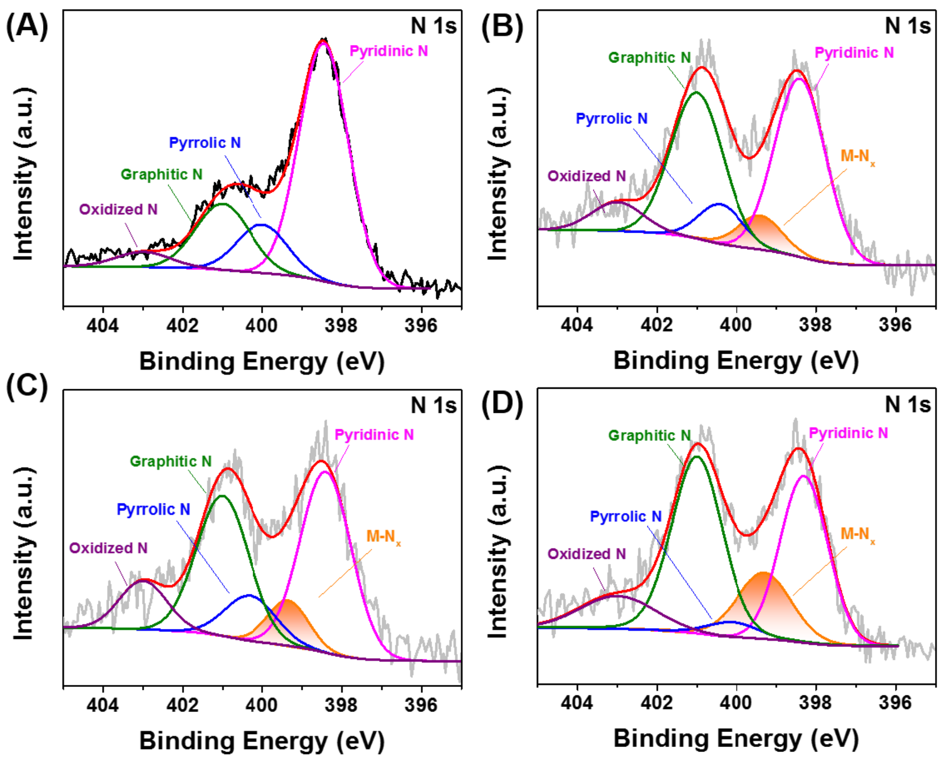

Fig. S13 XPS spectra of N 1s for (**A**) N-C, (**B**) Mo-N-C, (**C**) Fe-N-C and (**D**) MoFe-N-C

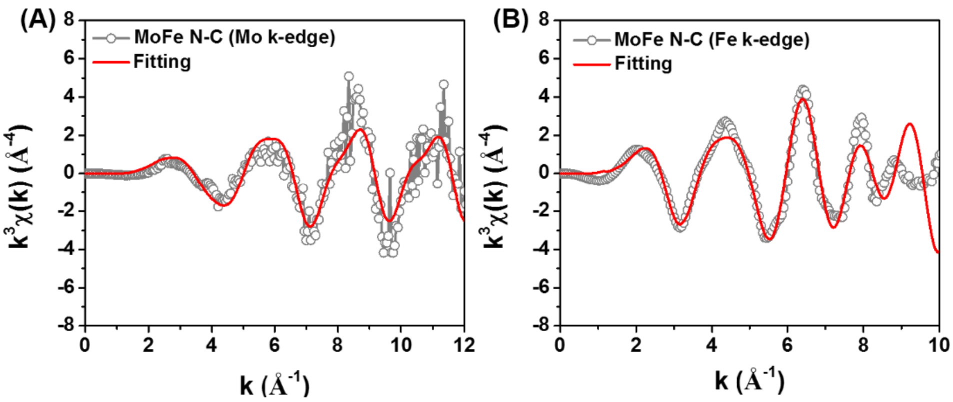

Fig. S14 The *k*-space EXAFS curve and corresponding fitting curve of MoFe-N-C at Mo K-edge (**A**) and Fe K-edge (**B**)

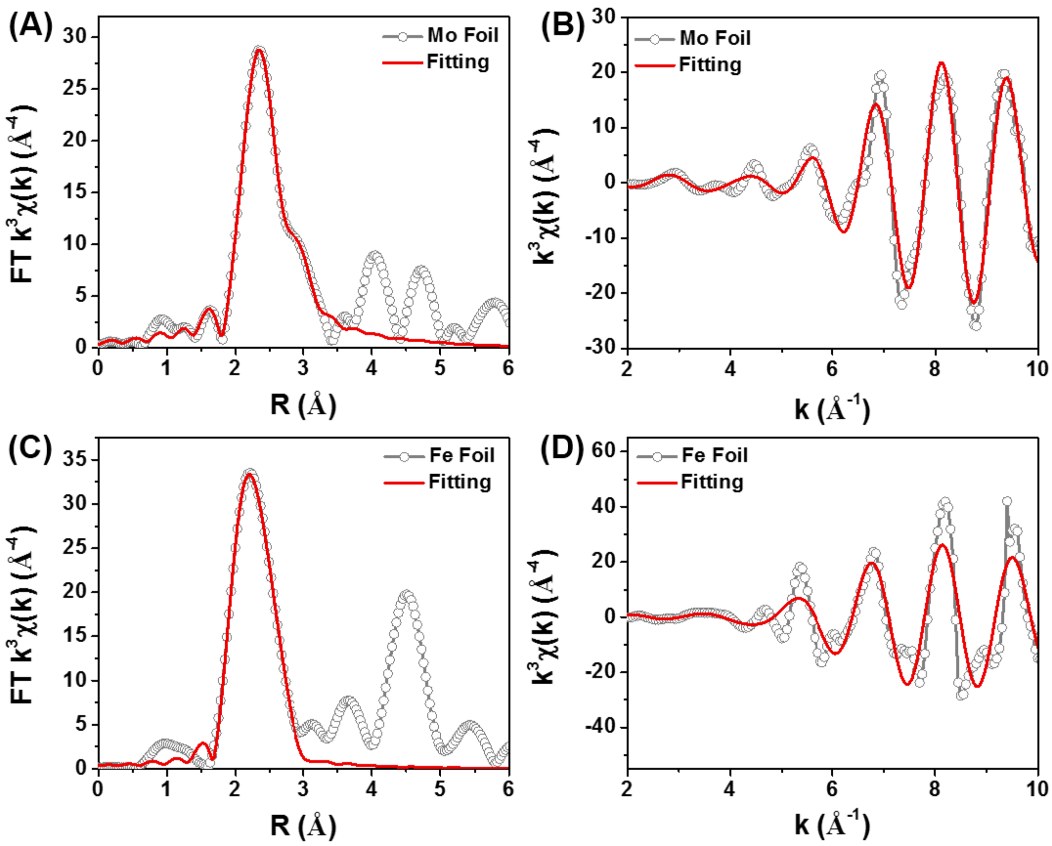

Fig. S15 (**A**) The *R*-space EXAFS curve and corresponding fitting curve of Mo Foil. (**B**) The *k*-space EXAFS curve and corresponding fitting curve of Mo Foil. (**C**) The *R*-space EXAFS curve and corresponding fitting curve of Fe Foil. (**D**) The *k*-space EXAFS curve and corresponding fitting curve of Fe Foil

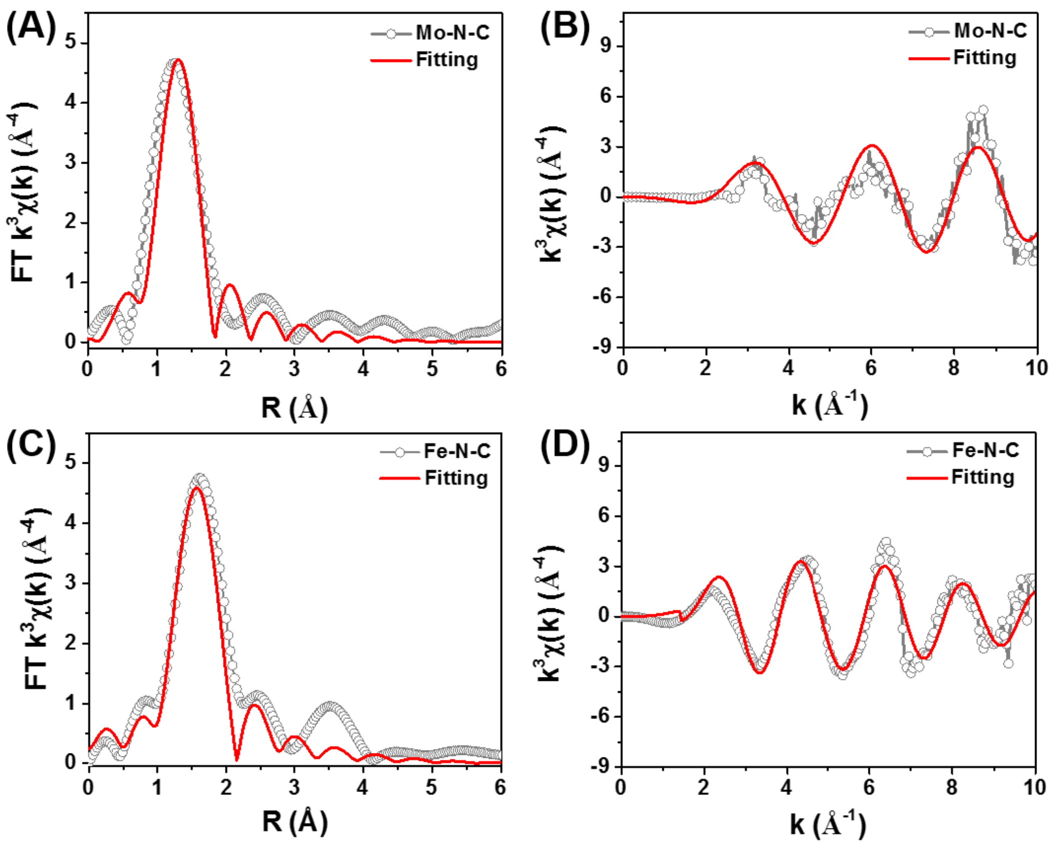

Fig. S16 (**A**) The *R*-space EXAFS curve and corresponding fitting curve of Mo-N-C. (**B**) The *k*-space EXAFS curve and corresponding fitting curve of Mo-N-C. (**C**) The *R*-space EXAFS curve and corresponding fitting curve of Fe-N-C. (**D**) The *k*-space EXAFS curve and corresponding fitting curve of Fe-N-C

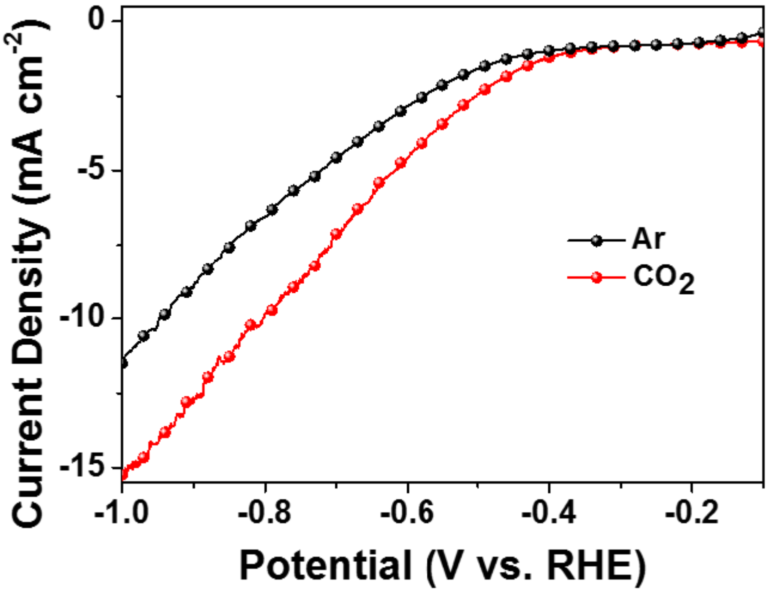

Fig. S17 LSV curve of MoFe-N-C in CO_2- or Ar-saturated 0.1 M $KHCO_3$ electrolyte

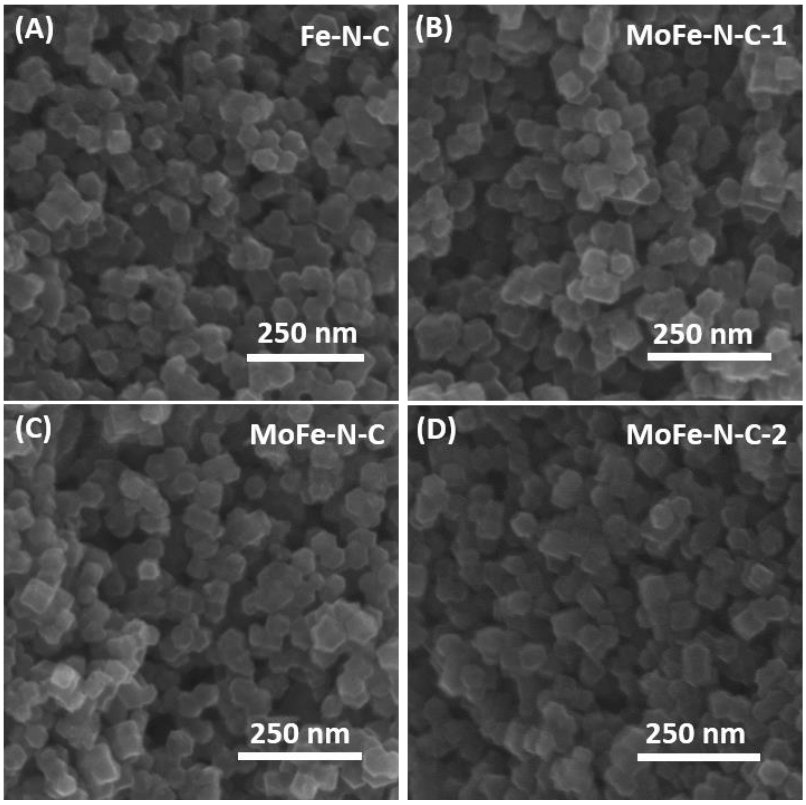

Fig. S18 SEM images of the samples with different Mo content. Their size and morphology hardly changed with the variation of Mo content

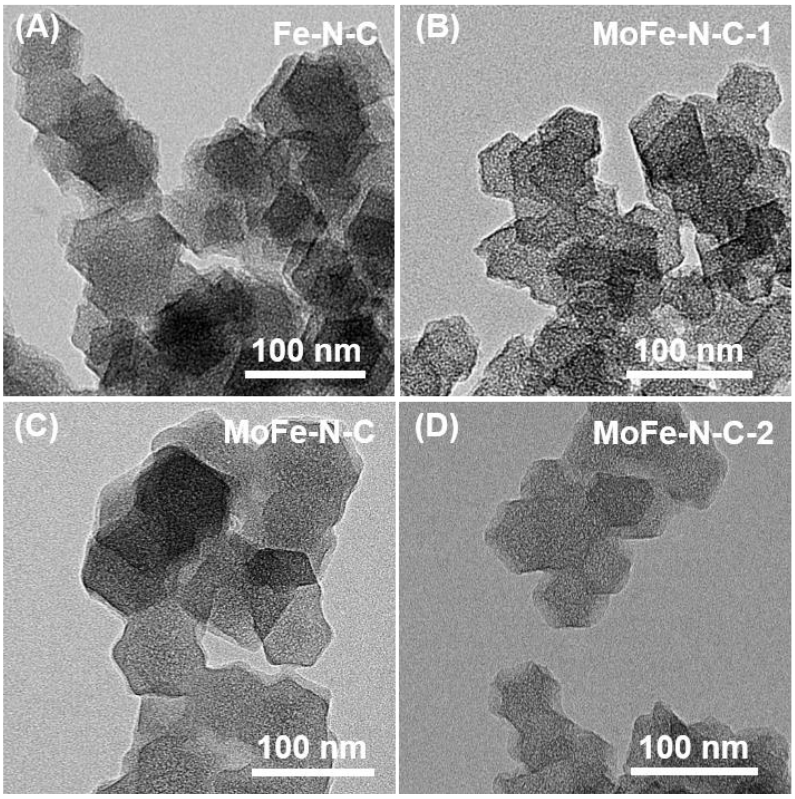

Fig. S19 HR-TEM images of the samples with different Mo content

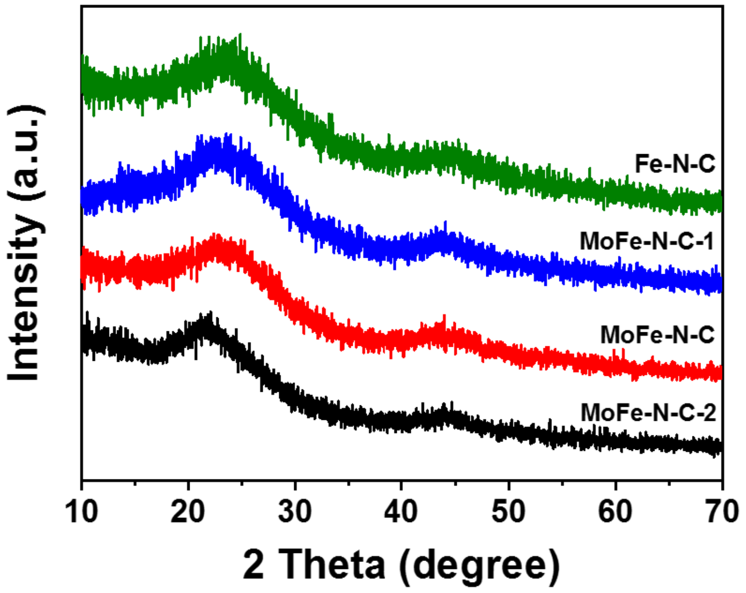

Fig. S20 The XRD spectra of the samples with different Mo content. Their crystal structure hardly changed with the variation of Mo content

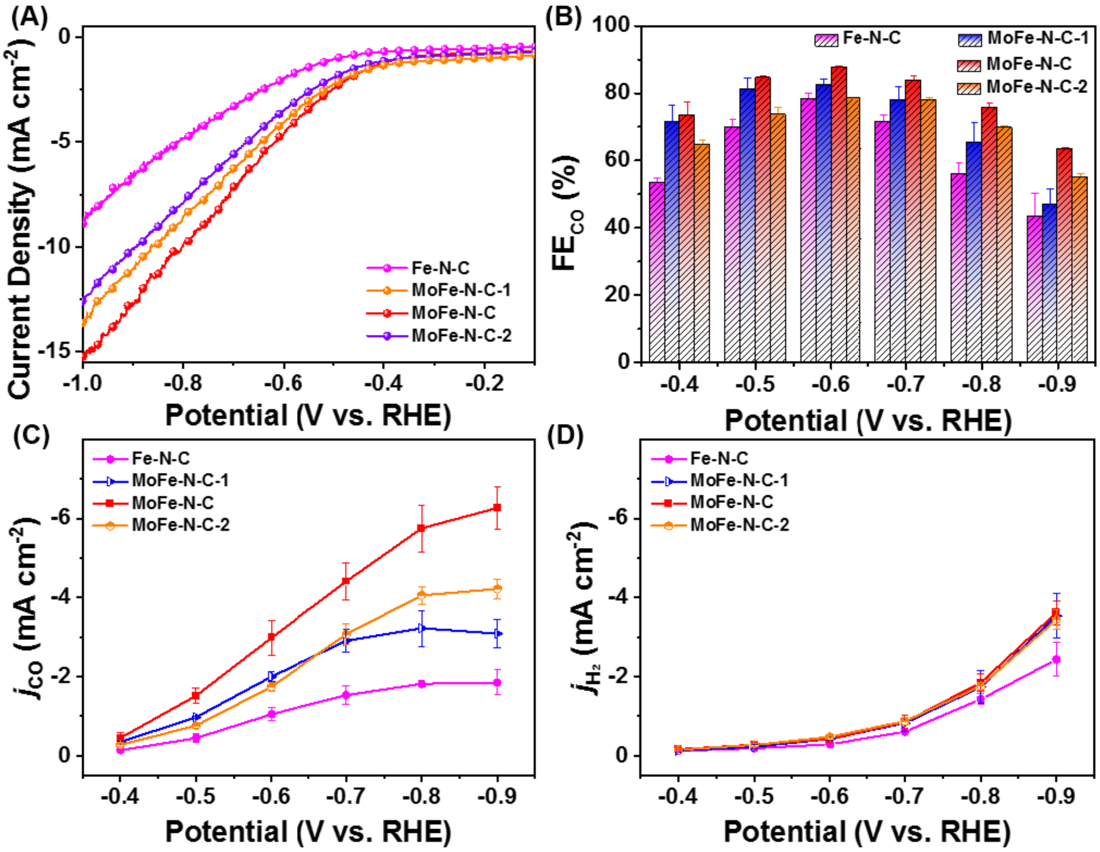

Fig. S21 (**A**) LSV curves of the catalysts measured in CO_2-saturated 0.1 $KHCO_3$ electrolyte. (**B**) CO Faradaic efficiency of the different catalysts at selected potentials. (**C**) CO and (**D**) H_2 partial current density of the different catalysts at selected potentials

Inductively coupled plasma-atomic emission spectroscopy (ICP-AES) was used to detect the metal content (Table S4 and S5).

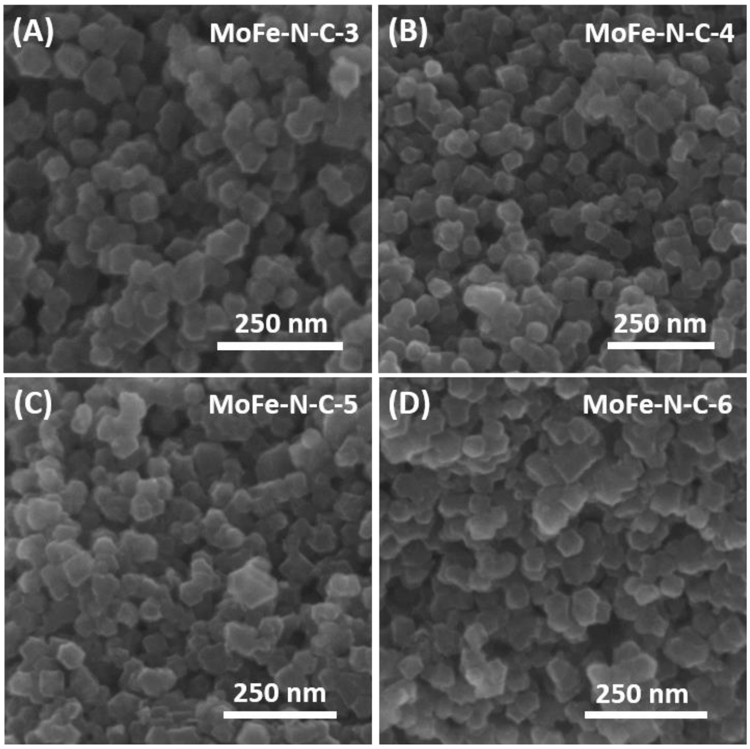

Fig. S22 SEM images of the samples with different Fe content. Their size and morphology were not affected by the Fe content

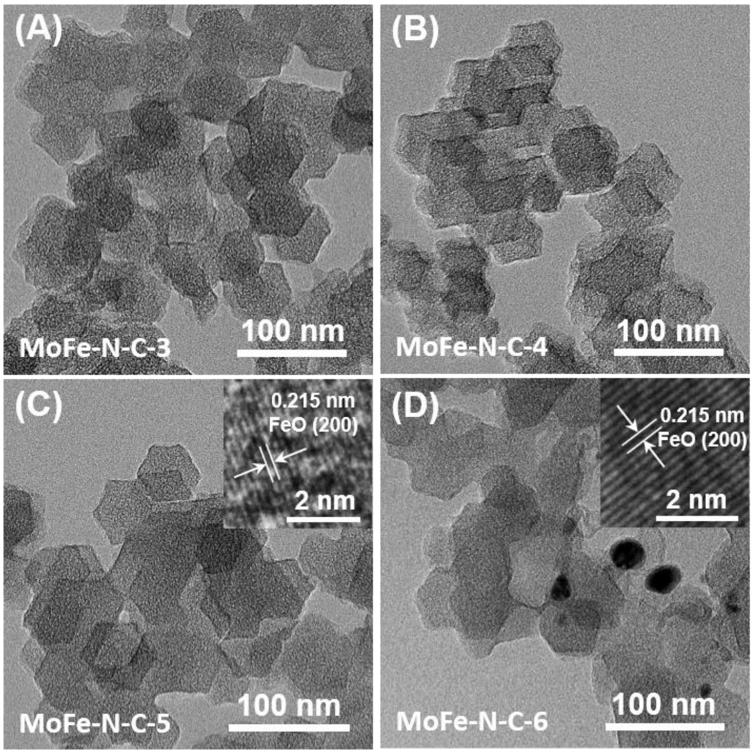

Fig. S23 HR-TEM images of the samples with different Fe content

When the input of Fe precursor exceeds a certain amount, the FeO nanoparticles were observed inside the material by HR-TEM, which were generated from excess Fe atoms and trace oxygen.

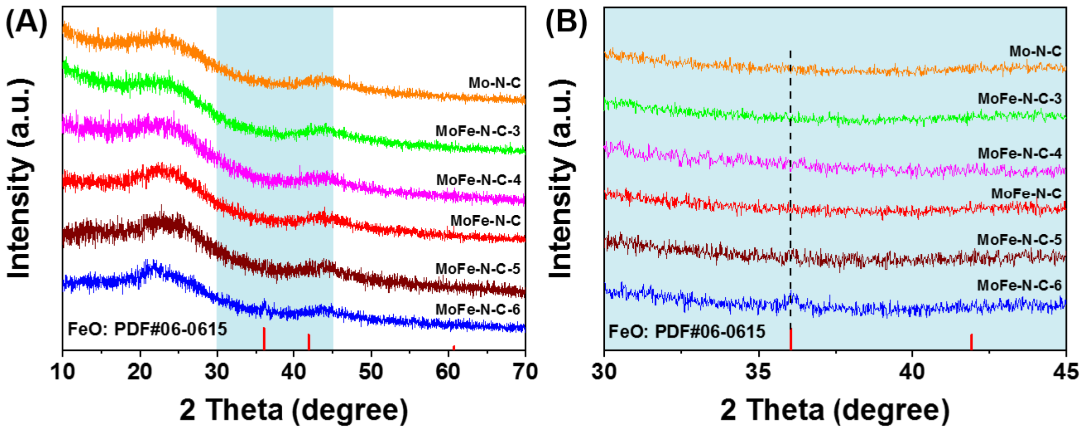

Fig. S24 (**A**) The XRD spectra of the samples with different Fe content. (**B**) The magnification of the blue area in (**A**)

When the input of Fe precursor exceeds a certain amount, the FeO nanoparticles were observed inside the material by XRD patterns.

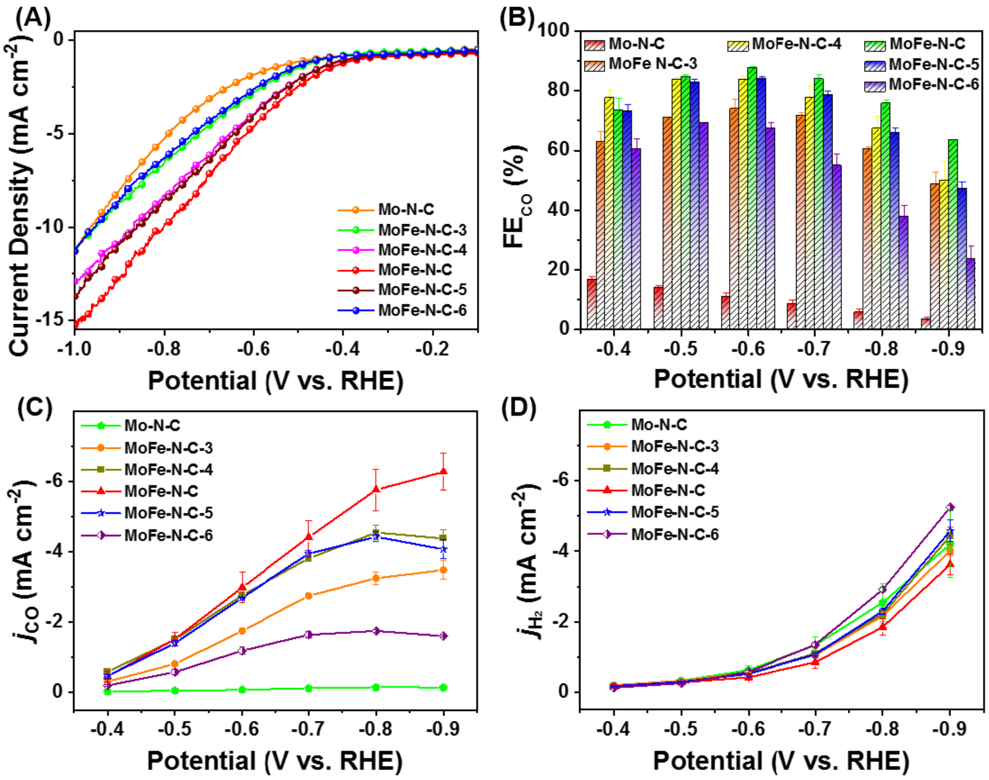

Fig. S25 (**A**) LSV curves of catalysts measured in CO_2-saturated 0.1 $KHCO_3$ electrolyte. (**B**) CO Faradaic efficiency of the different catalysts at selected potentials. (**C**) CO and (**D**) H_2 partial current density of the different catalysts at selected potentials

The catalytic activity and selectivity firstly promoted with the increase of Fe content. Whereas, excess Fe causes the agglomeration of metal atoms, which degrades its catalytic activity and selectivity.

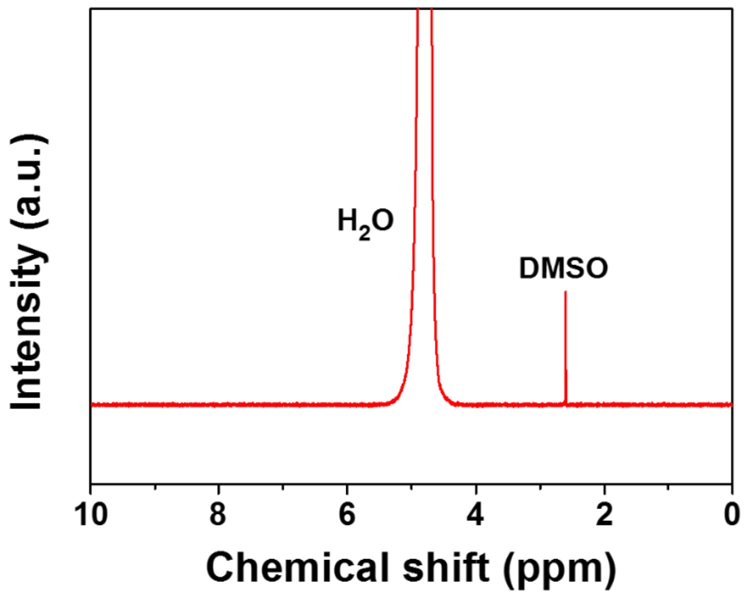

Fig. S26 The ^{1}H NMR spectrum of the electrolyte after CO_2RR electrolysis (at -0.60 V, 1h) by using MoFe-N-C catalyst

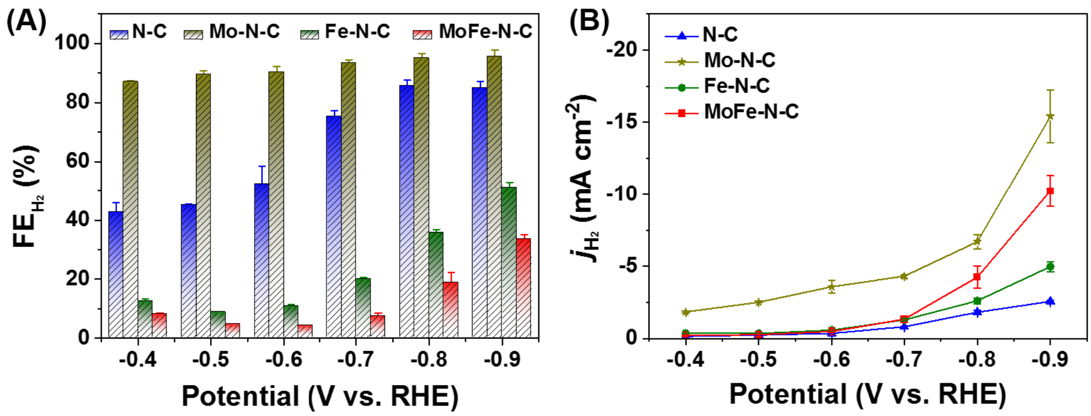

Fig. S27 Electrocatalytic Performance in the flow cell: (**A**) H_2 Faradaic efficiency of different catalysts at selected potentials. (**B**) H_2 partial current density of different catalysts at selected potentials

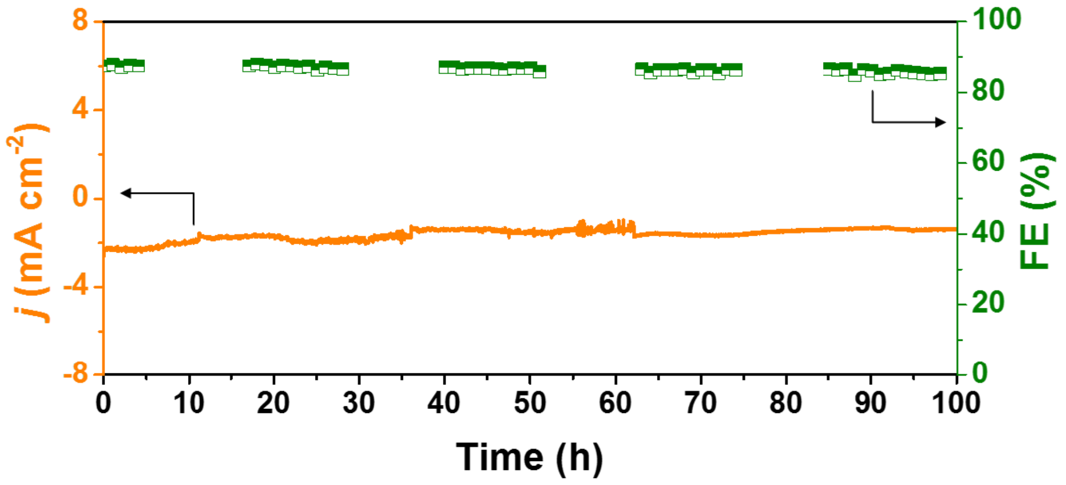

Fig. S28 Stability test of MoFe-N-C at -0.60 V for 100

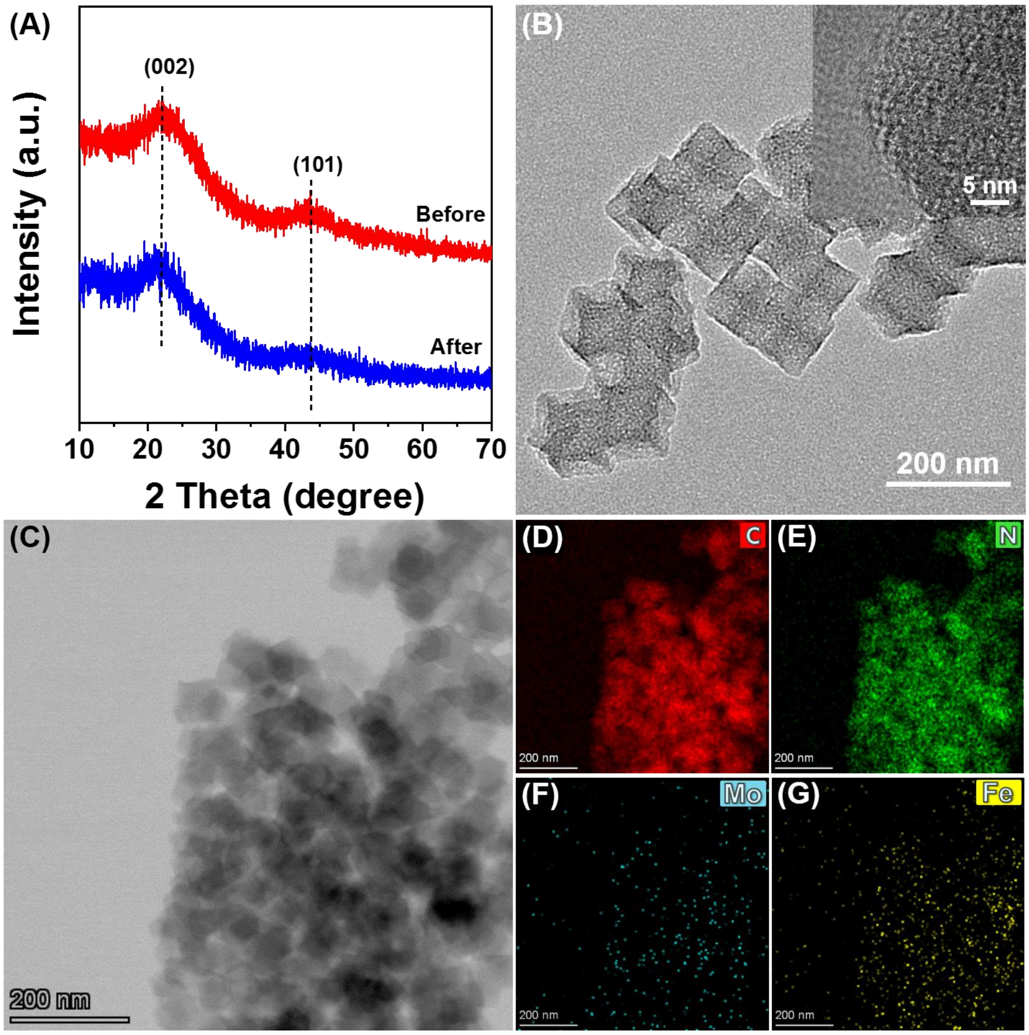

Fig. S29 (**A**) The XRD pattern of MoFe-N-C catalysts before and after potentiostatic electrolysis. (**B**) TEM and HR-TEM image of MoFe-N-C catalysts after potentiostatic electrolysis. (**C- G**) EDS mappings of MoFe-N-C catalysts after potentiostatic electrolysis

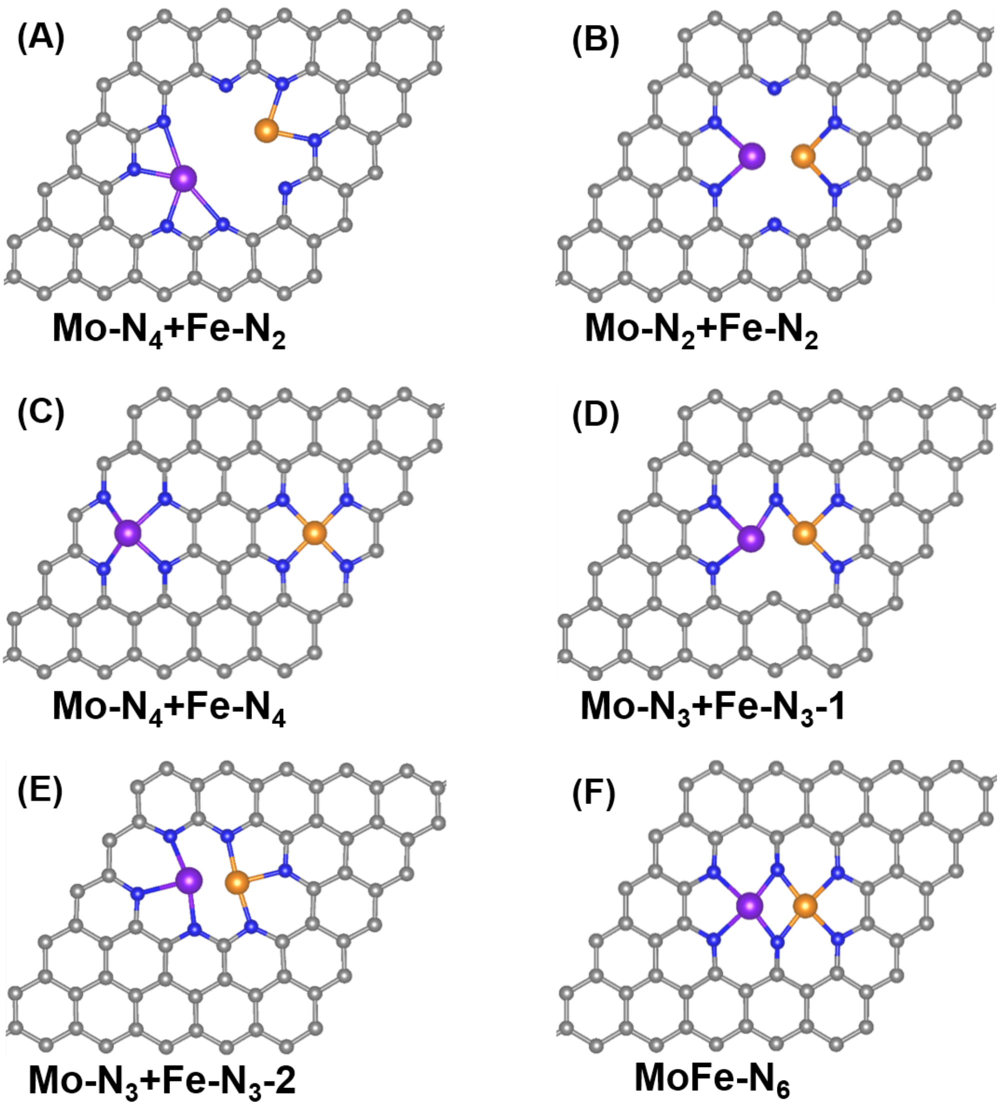

Fig. S30 Optimized geometry of the (**A**) Mo-N_4+Fe-N_2, (**B**) Mo-N_2+Fe-N_2, (**C**) Mo-N_4+Fe-N_4, (**D**) Mo-N_3+Fe-N_3-1, (**E**) Mo-N_3+Fe-N_3-2 and (**F**) MoFe-N_6. Purple, yellow, blue, red, white and gray represent the Mo, Fe, N, O, H and C atoms, respectively.

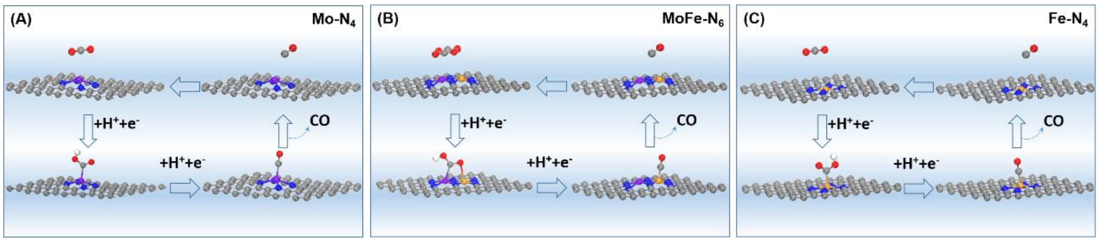

Fig. S31 Optimized catalytic models and reaction pathways of (**A**) Mo-N_4 sites, (**B**) MoFe-N_6 sites and (**C**) Fe-N_4 sites. Purple, yellow, blue, red, white and gray represent the Mo, Fe, N, O, H and C atoms, respectively.

The electrochemical CO_2RR process is composed of the following four steps: CO_2 adsorption (CO_2 + * → *COO), proton-coupled electron transfer (*COO + H^+ + e^- →

*COOH), further proton-coupled electron transfer (*COOH + H^+ + e^- → *CO + H_2O), and CO release (*CO → CO + *).

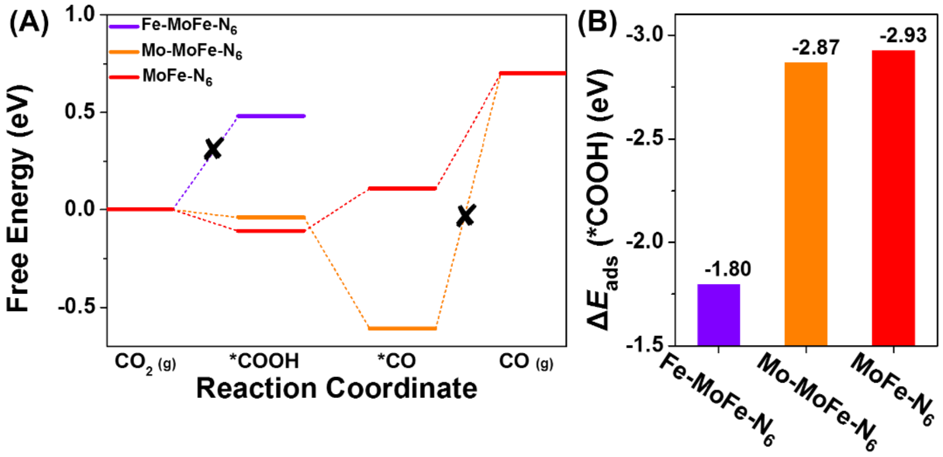

Fig. S32 (**A**) Free energy diagrams of different catalytic sites for CO_2RR. (**B**) Absorption energy of *COOH intermediates on different catalytic sites

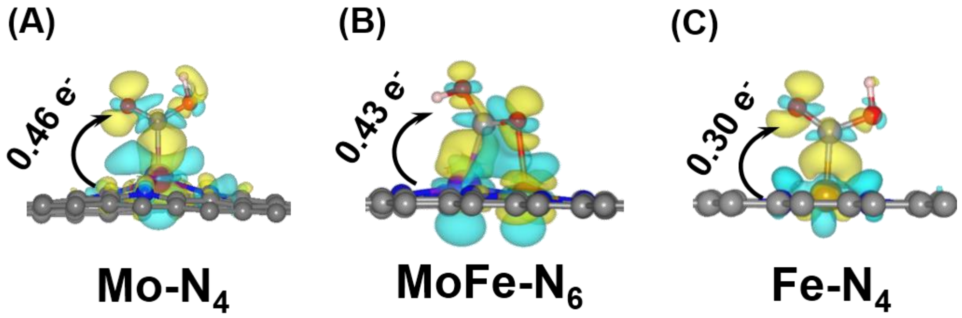

Fig. S33 Charge density difference distributions of (**A**) Mo-N_4 sites, (**B**) MoFe-N_6 sites and (**C**) Fe-N_4 sites with *COOH intermediates adsorbed. Yellow and blue areas denote charge accumulation and depletion, respectively.

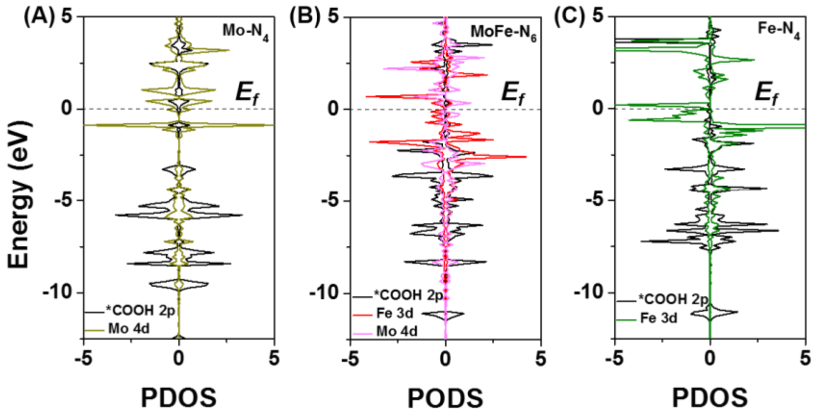

Fig. S34 Partial density of states for (**A**) Mo-N_4 sites, (**B**) MoFe-N_6 sites and (**C**) Fe-N_4 sites with *COOH intermediates adsorbed

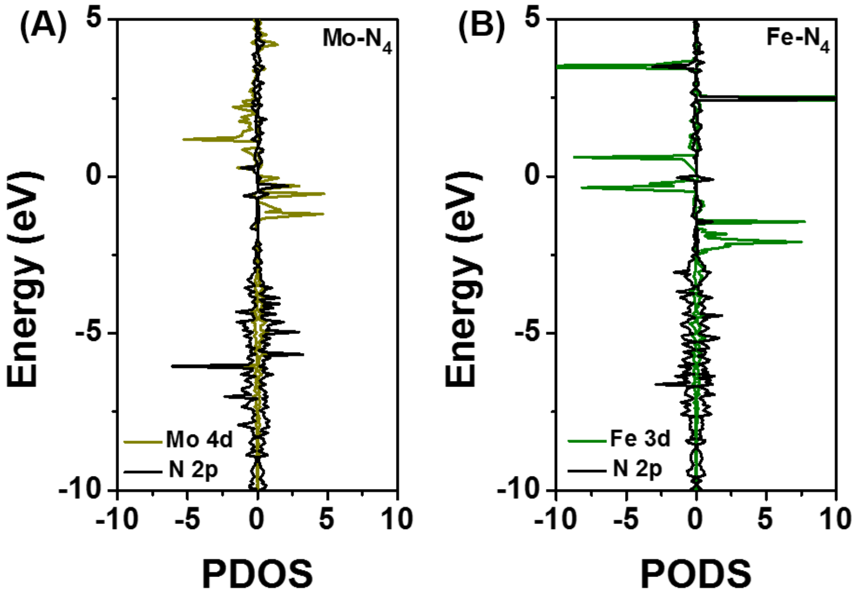

Fig. S35 Calculated partial density of states (PDOS) for (**A**) Mo-N_4 sites and (**B**) Fe-N_4 sites

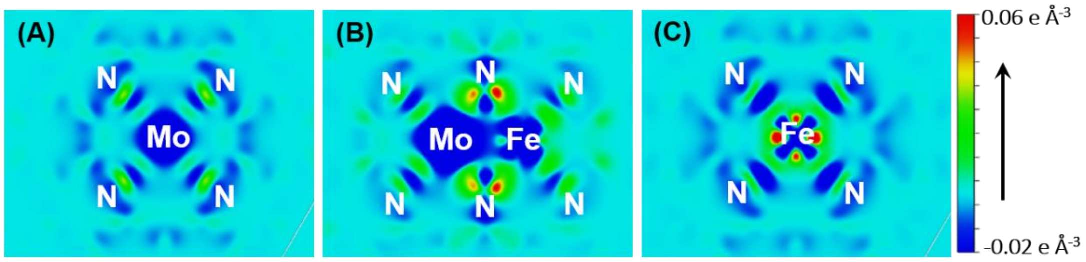

Fig. S36 Charge density difference distributions for (**A**) Mo-N_4 sites, (**B**) MoFe-N_6 sites and (**C**) Fe-N_4 sites, with a range from -0.02 e·Å^{-3} (blue) to +0.06 e·Å^{-3} (red)

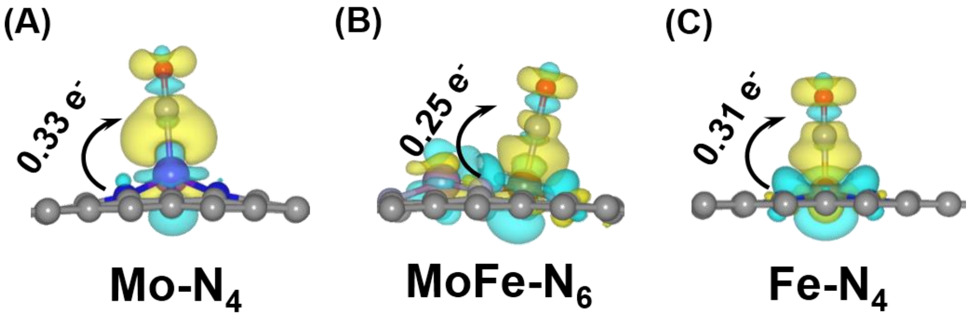

Fig. S37 Charge density difference distributions of (**A**) Mo-N_4 sites, (**B**) MoFe-N_6 sites and (**C**) Fe-N_4 sites with *CO intermediates adsorbed. Yellow and blue areas denote charge accumulation and depletion, respectively.

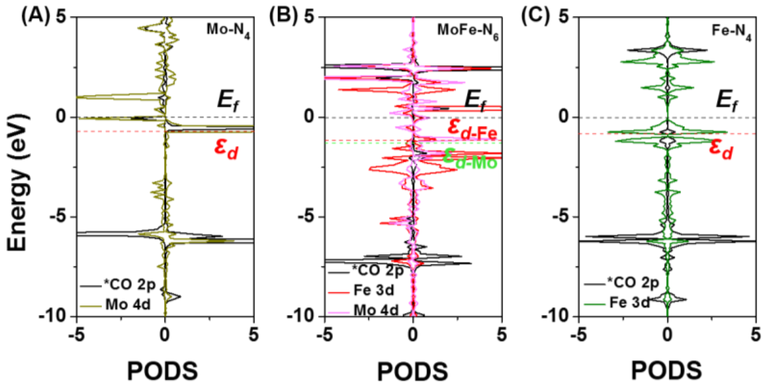

Fig. S38 Partial density of states for (**A**) Mo-N_4 sites, (**B**) MoFe-N_6 sites and (**C**) Fe-N_4 sites with *CO intermediates adsorbed

Notably, the d-band center of Fe (-1.15 eV) on MoFe-N_6 site is closer to the Fermi level than Mo (-1.20 eV), so the *CO intermediate for MoFe-N-C tends to adsorbed on Fe atoms.

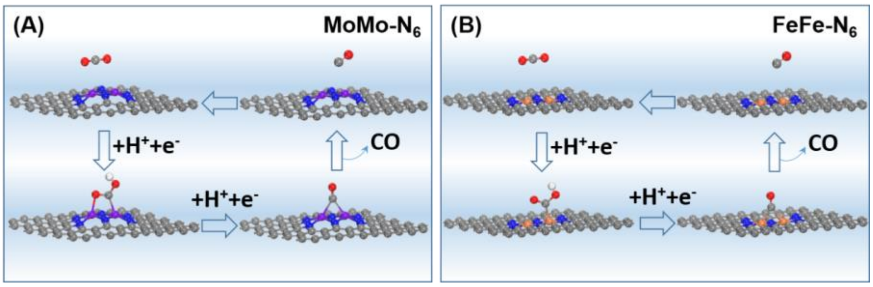

Fig. S39 Optimized catalytic models and reaction pathways of (**A**) MoMo-N_6 sites and (**B**) FeFe-N_6 sites. Purple, yellow, blue, red, white and gray represent the Mo, Fe, N, O, H and C atoms, respectively.

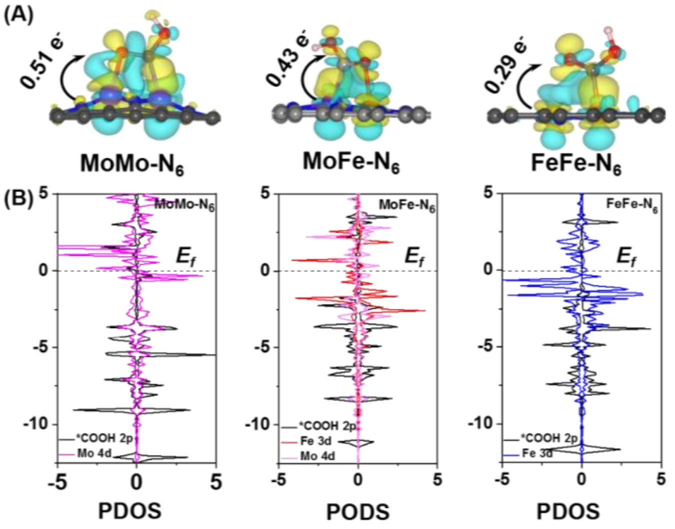

Fig. S40 (**A**) Charge density difference distributions of MoMo-N_6 sites, MoFe-N_6 sites and FeFe-N_6 sites with *COOH intermediates adsorbed. Yellow and blue areas denote charge accumulation and depletion, respectively. (**B**) Partial density of states for MoMo-N_6 sites, MoFe-N_6 sites and FeFe-N_6 sites with *COOH intermediates adsorbed, respectively.

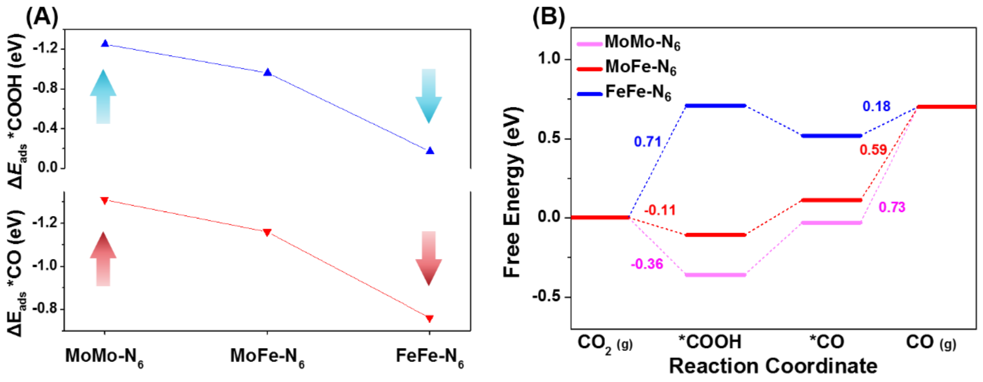

Fig. S41 (**A**) The absorption energy of the *COOH intermediates (up column) and *CO intermediates (bottom column) on different catalytic sites. (**B**) Free energy diagrams of the catalysts for CO_2RR

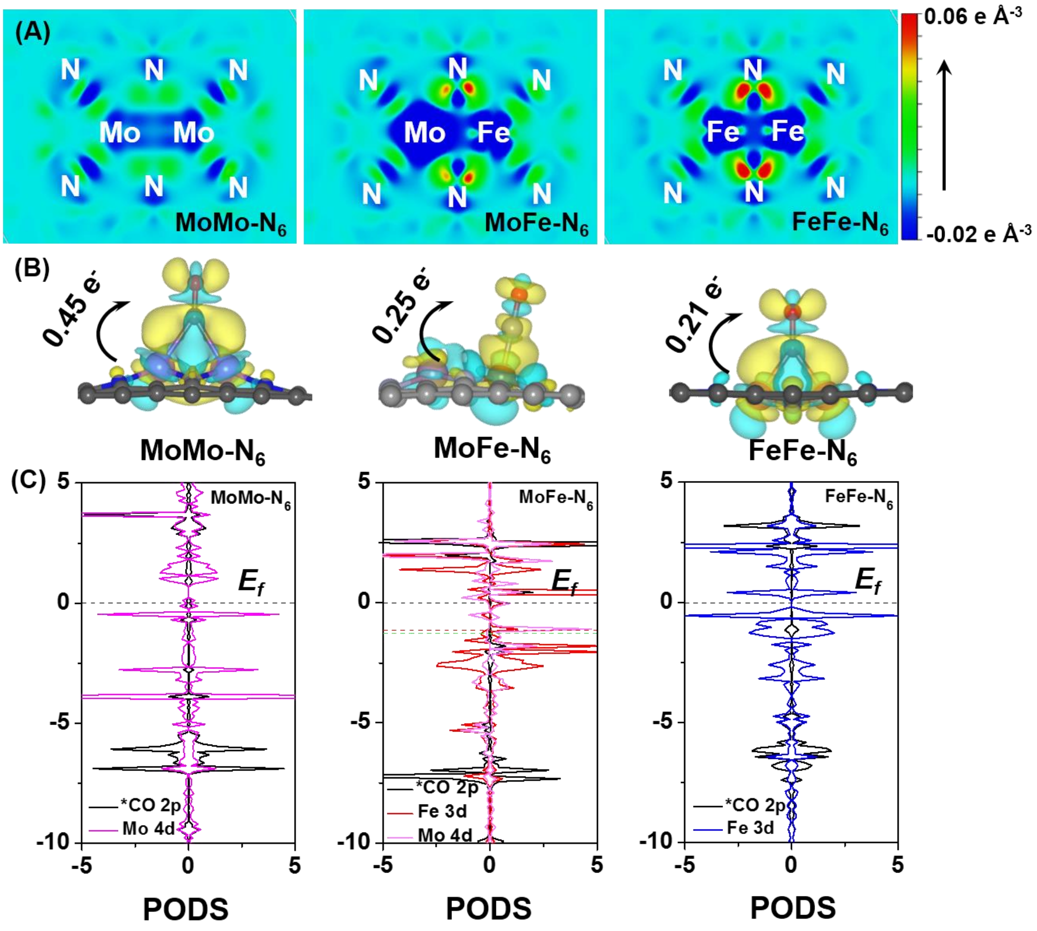

Fig. S42 (**A**) Charge density difference distributions for MoMo-N_6 sites, MoFe-N_6 sites and FeFe-N_6 sites, with a range from -0.02 e·$Å^{-3}$ (blue) to +0.06 e·$Å^{-3}$ (red). (**B**) Charge density difference distributions of MoMo-N_6 sites, MoFe-N_6 sites and FeFe-N_6 sites with *CO intermediates adsorbed. Yellow and blue areas denote charge accumulation and depletion, respectively. (**C**) Partial density of states for MoMo-N_6 sites, MoFe-N_6 sites and FeFe-N_6 sites with *CO intermediates adsorbed

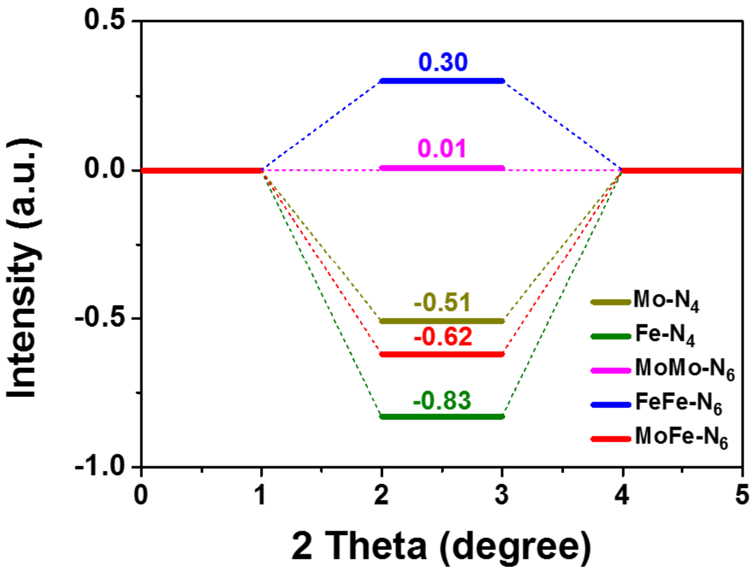

Fig. S43 Free energy diagram for HER on Mo-N_4 sites, MoMo-N_6, MoFe-N_6 sites, FeFe-N_6 and Fe-N_4 sites at U = 0 V versus RHE

Table S1 The XPS peak fitting results of N-C, Mo-N-C, Fe-N-C and MoFe-N-C

	**N-C**	**Mo-N-C**	**Fe-N-C**	**MoFe-N-C**		
Region	BE [eV]	BE [eV]	BE [eV]	BE [eV]	Assigned to	Refs
**C 1s**	284.52	284.52	284.52	284.52	C-C	[S11, S12]
	285.30	285.30	285.30	285.30	C-N	[S13]
	288.00	288.00	288.00	288.00	O-C=O	[S12]
**N 1s**	398.40	398.40	398.40	398.40	Pyridinic N	[S14]
	399.40	399.40	399.40	-	M-N_x	[S12, S15]
	400.30	400.40	400.40	400.40	Pyrrolic N	[S11]
	401.10	401.00	401.00	401.00	Graphitic N	[S8, S15]
	403.00	403.00	403.00	403.00	Oxidized N	[S15, S16]

Table S2 Summary of elemental quantification determined by XPS for different catalysts

		**N-C**	**Mo-N-C**	**Fe-N-C**	**MoFe-N-C**
**Region**	**Assigned to**	**Species amount (%)**			
**C 1s**	C-C	53.19	54.27	52.21	54.76
	C-N	40.43	42.70	40.71	41.29
	O-C=O	6.38	3.03	7.08	3.95
**N 1s**	Pyridinic N	62.98	48.74	39.00	32.56
	M-N_x	0	6.79	9.00	15.50
	Pyrrolic N	13.26	12.54	10.00	3.10
	Graphitic N	19.34	24.69	32.00	38.76
	Oxidized N	4.42	7.24	10.00	10.08

Table S3 EXAFS fitting parameters at the Fe and Mo K-edge. (S_0^2=0.55, 0.98)

Sample	Path	C.N.	R (Å)	$\sigma^2\times10^3$ (Å^2)	ΔE (eV)	R factor
Fe foil	Fe-Fe	8*	2.45±0.01	2.1±.5	2.8±2.2	0.009
	Fe-Fe	6*	2.85±0.01	1.2±1.9	5.4±3.7	
Fe-N-C	Fe-N	3.9±0.4	2.04±0.03	0.1±0.1	4.9±1.0	0.012
MoFe-N-C	Fe-N	4.2±1.7	1.97±0.02	3.5±4.2	2.2±3.0	0.013
	Fe-Mo	1.6±1.0	2.35±0.02	2.7±5.6	5.4±3.8	
Mo foil	Mo-Mo	8*	2.72±0.01	3.7±0.3	-7.8±0.8	0.001
	Mo-Mo	6*	3.14±0.01	3.5±0.4	-4.6±1.4	
Mo-N-C	Mo-N	3.6±2.8	1.70±0.06	2.5±5.6	-8.1±29.6	0.079
MoFe-N-C	Mo-N	3.5±2.5	1.68±0.04	4.3±3.4	-25.0±18.6	0.035
	Mo-Fe	0.4±0.2	2.41±0.05	-0.3±5.8	21.9±13.3	

C.N.: coordination numbers; *R*: bond distance; σ^2: Debye-Waller factors; Δ*E*: the inner potential correction. *R* factor: goodness of fit. * fitting with fixed parameter.

Table S4 Mo and Fe content in the catalysts obtained by ICP measurements

Sample	Mo (wt%)	Fe (wt%)
Fe-N-C	--	0.48
MoFe-N-C-1	0.09	0.56
MoFe-N-C	0.21	0.52
MoFe-N-C-2	0.43	0.60

Table S5 Mo and Fe content in the catalysts obtained by ICP measurements

Sample	Mo (wt%)	Fe (wt%)
Mo-N-C	0.21	--
MoFe-N-C-3	0.24	0.21
MoFe-N-C-4	0.21	0.41
MoFe-N-C	0.21	0.52
MoFe-N-C-5	0.22	0.61
MoFe-N-C-6	0.19	0.62

Table S6 The comparison of MoFe N-C with other reported atomically dispersed Fe-based CO_2RR electrocatalysts

Sample	Electrolyte	Potential (V vs. RHE)[a]	FE_{CO} (%)[b]	TOF (h^{-1})	Refs.
**MoFe-N-C**	0.5 M $KHCO_3$	-0.6	95.96	3,336	This work
**Fe^{3+}-N-C**	0.5 M $KHCO_3$	-0.45	90	~1,500	Science, 2019, 364, 1091-1094.
**ZIF-NC-Ni-Fe**	0.1 M $KHCO_3$	-0.6	97.8	2210	Angew. Chem. Int. Ed., 2022, 61, e202205632.
**NiFe-DASC**	0.5 M $KHCO_3$	−0.8	94.5	-	Nat. Commun., 2021, 12, 4088.
**Fe-N-C**	0.1 M $KHCO_3$	-0.6	74	-	Chem. Eng. J., 2020, 389, 124323
**edge-Fe−N_4**	0.5 M $KHCO_3$	-0.5	93.5	-	ACS Energy Lett., 2019, 4, 1778−1783.
**Fe-N-C-1000**	0.1 M $KHCO_3$	-0.5	~90	~2600	Angew. Chem. Int. Ed., 2021, 60, 1022–1032.
**Fe_1-NSC**	0.5 M $KHCO_3$	-0.48	98.6	1197	Angew. Chem. Int. Ed., 2022, 61, e202206233.
**CoPc©Fe-N-C**	0.5 M KOH	-0.84	93	-	Adv. Mater., 2019, 31, 1903470.
**DNG-SAFe**	1.0 M KOH	-0.69	89	-	Adv. Mater., 2021, 33, 2003238.
**Fe/NG-750**	0.1 M $KHCO_3$	-0.57	~80	-	Adv. Energy Mater., 2018, 8, 1703487.
**Fe-N-G-p**	0.1 M $KHCO_3$	-0.58	94	1630	ACS Catal., 2020, 10, 19, 10803–10811.
**Fe_1-N-C**	0.5 M $KHCO_3$	-0.5	86.5	~2800	Angew. Chem. Int. Ed., 2020, 59, 20589.

(Note: [b] is the optimal CO Faraday efficiency under the potential [a].)

Table S7 Comparison of total energy, formation energy and the distance between Mo and Fe atoms of each structure

Configuration	E/eV
Mo-N_4+Fe-N_2	-598.17
Mo-N_2+Fe-N_2	-618.96
Mo-N_4+Fe-N_2	-637.38
Mo-N_3+Fe-N_3-1	-639.76
Mo-N_3+Fe-N_3-2	-630.14
MoFe-N_6	-642.27

Supplementary References

[S1] J. Tang, X. Zhao, J. Li, R. Guo, Y. Zhou et al., Gallium-Based Liquid Metal Amalgams: Transitional-State Metallic Mixtures with Enhanced and Tunable Electrical, Thermal, and Mechanical Properties. ACS Appl. Mater. Interfaces **9**, 35977-35987 (2017). https://doi.org/10.1021/acsami.7b10256

[S2] G. Kresse, D. Joubert, From Ultrasoft Pseudopotentials to the Projector Augmented-Wave Method. Phys. Rev. B **59**, 1758-1775 (1999). https://doi.org/10.1103/PhysRevB.59.1758

[S3] J.P. Perdew, K. Burke, M. Ernzerhof, Ernzerhof, M. Generalized Gradient Approximation Made Simple. Phys. Rev. Lett. **77**, 3865-3868 (1996). https://doi.org/10.1103/PhysRevLett.77.3865

[S4] S. Grimme, Semiempirical Gga-Type Density Functional Constructed with a Long-Range Dispersion Correction. J. Comput. Chem. **27**, 1787-1799 (2006). https://doi.org/10.1002/jcc.20495

[S5] H. J. Monkhorst, J. D. Pack, Special Points for Brillouin-Zone Integrations. Phys. Rev. B **13**, 5188-5192 (1976). https://doi.org/10.1103/PhysRevB.13.5188

[S6] J. K. Nørskov, J. Rossmeisl, A. Logadottir, L. Lindqvist, J.R. Kitchin et al., Origin of the Overpotential for Oxygen Reduction at a Fuel-Cell Cathode. J. Phys. Chem. B **108**, 17886-17892 (2004). https://doi.org/10.1021/jp047349j

[S7] J. Rossmeisl, A. Logadottir, J. K. Nørskov, Electrolysis of Water on (Oxidized) Metal Surfaces. Chem. Phys. **319**, 178-184 (2005). https://doi.org/10.1016/j.chemphys.2005.05.038

[S8] J. Li, M. Chen, D.A. Cullen, S. Hwang, M. Wang et al., Atomically Dispersed Manganese Catalysts for Oxygen Reduction in Proton-Exchange Membrane Fuel Cells. Nat. Catal. **1**, 935-945 (2018). https://doi.org/10.1038/s41929-018-0164-8

[S9] L. Lajaunie, F. Boucher, R. Dessapt, P. Moreau, Quantitative Use of Electron Energy-Loss Spectroscopy Mo-$M_{2,3}$ Edges for the Study of Molybdenum Oxides. Ultramicroscopy **149**, 1-8 (2015). https://doi.org/10.1016/j.ultramic.2014.11.002

[S10] Z. Zeng, L.Y. Gan, H. B. Yang, X. Su, J. Gao et al., Orbital Coupling of Hetero-Diatomic Nickel-Iron Site for Bifunctional Electrocatalysis of CO_2 Reduction and Oxygen Evolution. Nat. Commun. **12**, 4088 (2021). https://doi.org/10.1038/s41467-021-24052-5

[S11] Y. Wang, Z. Zhang, G. Jia, L. Zheng, J. Zhao et al., Elucidating the Mechanism of the Structure-Dependent Enzymatic Activity of Fe–N/C

Oxidase Mimics. Chem. Commun. **55**, 5271-5274 (2019). https://doi.org/10.1039/C9CC01503E

[S12] W. Liu, L. Zhang, X. Liu, X. Liu, X. Yang et al. Discriminating Catalytically Active Fenx Species of Atomically Dispersed Fe–N–C Catalyst for Selective Oxidation of the C–H Bond. J. Am. Chem. Soc. **139**, 10790-10798 (2017). https://doi.org/10.1021/jacs.7b05130

[S13] J. Pei, T. Wang, R. Sui, X. Zhang, D. Zhou et al., N-Bridged Co–N–Ni: New Bimetallic Sites for Promoting Electrochemical CO_2 Reduction. Energy Environ. Sci. **14**, 3019-3028 (2021). https://doi.org/10.1039/D0EE03947K

[S14] Y. Wang, K. Qi, S. Yu, G. Jia, Z. Cheng et al., Revealing the Intrinsic Peroxidase-Like Catalytic Mechanism of Heterogeneous Single-Atom Co–MoS_2. Nano-Micro Lett. **11**, 102 (2019). https://doi.org/10.1007/s40820-019-0324-7

[S15] Y. Wang, B. J. Park, V. K. Paidi, R. Huang, Y. Lee et al., Precisely Constructing Orbital Coupling-Modulated Dual-Atom Fe Pair Sites for Synergistic CO_2 Electroreduction. ACS Energy Lett. **7**, 640-649 (2022). https://doi.org/10.1021/acsenergylett.1c02446

[S16] H. Cheng, X. Wu, M. Feng, X. Li, G. Lei et al., Atomically Dispersed Ni/Cu Dual Sites for Boosting the CO_2 Reduction Reaction. ACS Catal. **11**, 12673-12681 (2021). https://doi.org/10.1021/acscatal.1c02319
